# Metagenomic Investigation of the Short-Term Temporal and Spatial Dynamics of the Bacterial Microbiome and the Resistome Downstream of a Wastewater Treatment Plant in the Iskar River in Bulgaria

**DOI:** 10.3390/microorganisms12061250

**Published:** 2024-06-20

**Authors:** Deyan Donchev, Ivan N. Ivanov, Ivan Stoikov, Monika Ivanova

**Affiliations:** 1 National Reference Laboratory for Control and Monitoring of Antimicrobial Resistance, Department of Microbiology, National Center of Infectious and Parasitic Diseases, 26 Yanko Sakazov Blvd., 1504 Sofia, Bulgaria; 2 Paralax Life Sciences, Sofia Center, 47 Bacho Kiro Str., 1202 Sofia, Bulgaria

**Keywords:** metagenomics, freshwater river, resistance genes, carbapenemase, WWTP, sequencing

## Abstract

Waste Water Treatment Plants (WWTP) aim to reduce contamination in effluent water; however, studies indicate antimicrobial resistance genes (ARGs) persist post-treatment, potentially leading to their spread from human populated areas into the environment. This study evaluated the impact of a large WWTP serving 125,000 people on the Iskar River in Bulgaria, by characterizing the spatial and short-term temporal dynamics in bacterial community dynamics and resistance profiles of the surface water. Pairs of samples were collected biweekly on four dates from two different locations, one about 800 m after the WWTP effluents and the other 10 km downstream. Taxonomic classification revealed the dominance of *Pseudomonodota* and *Bacteriodota*, notably the genera *Flavobacterium*, *Aquirufa*, *Acidovorax*, *Polynucleobacter*, and *Limnohabitans*. The taxonomic structure corresponded with both lentic and lotic freshwater habitats, with *Flavobacterium* exhibiting a significant decrease over the study period. Principal Coordinate Analysis revealed statistically significant differences in bacterial community composition between samples collected on different dates. Differential abundance analysis identified notable enrichment of *Polynucleobacter* and *Limnohabitans.* There were shifts within the enriched or depleted bacterial taxa between early and late sampling dates. High relative abundance of the genes *erm(B)*, *erm(F)*, *mph(E)*, *msr(E)* (macrolides); *tet(C)*, *tet(O)*, *tet(W)*, *tet(Q)* and *tet(X)* (tetracyclines); *sul1* and *sul2* (sulphonamides); and *cfxA3*, *cfxA6* (beta-lactams) were detected, with trends of increased presence in the latest sampling dates and in the location closer to the WWTP. Of note, genes conferring resistance to carbapenems *bla*OXA-58 and *bla*IMP-33-like were identified. Co-occurrence analysis of ARGs and mobile genetic elements on putative plasmids showed few instances, and the estimated human health risk score (0.19) according to MetaCompare2.0 was low. In total, 29 metagenome-assembled genomes were recovered, with only a few harbouring ARGs. This study enhances our understanding of freshwater microbial community dynamics and antibiotic resistance profiles, highlighting the need for continued ARGs monitoring.

## 1. Introduction

The 21st century has witnessed the rise of bacterial antimicrobial resistance (AMR), wherein bacterial evolution results in a diminished effectiveness of antimicrobial drugs, posing a significant threat to public health by compromising treatment options for critical infections. The World Health Organization (WHO) singles out antibiotic resistance as one of the foremost threats to the cohesion of modern society [1,2]. The latest estimates suggest that the health burden of antibiotic-resistant bacterial infections is now comparable to that of influenza, tuberculosis and HIV/AIDS combined [3]. Approximately 1.27 million deaths are attributed to bacterial AMR per year, compared to the ideal scenario where all infections are caused by susceptible bacteria [4].

The environment plays a significant role in the development and dissemination of antimicrobial resistance bacteria (ARB) and antimicrobial resistance genes (ARG). Polluted waters, soils and other diverse ecological niches in the environment act as a meeting point for bacterial interchange of resistance genes, known as horizontal gene transfer (HGT) [5], and also as a vehicle of transmission [6]. Therefore, exploring AMR in the environment is another key tool to study both the regional and global AMR state and discover new resistance genes, and the functional shotgun metagenomic analysis is one of the few techniques available for this purpose [7,8]. 

Human survival and development in the modern world are intricately connected to the diverse ecosystem services offered by aquatic ecosystems, including food, water, and energy [9]. However, as crucial as they are, they serve as hot spots for the acquisition and dissemination of ARB and ARG and have received widespread attention due to their potential risks. Wastewater purification by Waste Water Treatment Plants (WWTP) in cities is heavily utilized to reduce contamination in the effluent water to surrounding aquatic and soil ecosystems and is regarded as the key link in mediating and mitigating the impact of urban pollution on natural environments. Numerous studies highlight that AMR is not completely eliminated during this process [10], and in fact WWTPs are among the main distributors of ARB and ARG into the environment [11,12]. As such, WWTPs are constantly being studied and monitored to display the complex relationships between bacteria and AMR occurring at those sites.

The Global Wastewater Project was the first to conduct shotgun metagenomic studies in wastewater in 101 countries, demonstrating the current AMR situation across the world [6]. The study was focused mainly on establishing the point-prevalence of ARGs in the effluents from major cities prior to WWTPs. More studies have also reported ARGs in additional sources such as sediment, soil and air [13,14,15]. Although the scientific literature and data on ARGs in effluent waters from WWTPs or in freshwater ecosystems such as lakes, dams or reservoirs is increasing, little is known about the changes that occur in the microbiome and the dynamics of ARGs in Bulgarian freshwater ecosystems.

The Iskar River flows entirely within the territory of Bulgaria. The average annual flow of the Iskar River is 54.5 m³/s. First, it services the municipality of Samokov, where the WWTP Samokov is located, which covers an area of 125,000 equivalent inhabitants, has a maximum capacity of 16,060,000 m^3^/year, but purifies only on the basis of mechanical and biological treatment. The treated water is discharged into the river, thus becoming the first major pollution source of ARG and ARB. Just 10 km further downstream, the Iskar River feeds the 675 million m³ artificially constructed Iskar Dam, which is a popular place for water recreation and is also a major source of water supply and electricity generation for the capital Sofia and the surrounding settlements. As such, the Iskar River in this precise region is of economic importance. Currently, there are no published taxonomic or AMR data on the Iskar River. Additionally, in 2022 a multi-institutional project “Establishment of the Iskar River Basin Water Management System” (IRWMS) as the first phase of the National Real-Time Water Management System (NRWMS) commenced. This project aims primarily to study flood risk prevention and management and adverse effects on human health and the environment by centralized collection, processing and analysis of information in real time, which is in accordance with European Parliament directive 2007/60 [16]. In the current study, we collected composite surface water samples from two locations in a ”time-series” manner along the Iskar River. The locations were strategically selected to reflect the dynamics of the river bacterial microbiome and the ARGs abundance immediately after the WWTP of Samokov city and prior to the Iskar Dam. We were able to demonstrate ARGs of clinical importance that were either located on putative plasmids or in close proximity to mobile genetic elements (MGE), thus with a potential for dissemination. In addition, there was significant evidence of higher ARG abundance in the sampling location closer to the WWTP compared to the downstream location, indicating the potential role of the WWTP in shaping the river resistome.

## 2. Methods

### 2.1. Samples

In total, eight composite water samples (1 L each) were collected in pairs along the Iskar River from the two locations (42.367698, 23.555463—Dragushinovo Village and 42.431095, 23.531900—villa area “Mechkata”) with an automatic sampler over a period of 24 hrs to avoid day-night bias on the following four dates: 3 November 2022, 17 November 2022, 8 December 2022, and 22 December 2022. This resulted in a total of 8 samples (2 locations × 4 dates). The first site was located about 800 m after the municipal WWTP in Samokov, thus being suitable for monitoring the effluent waters immediately after entering the river, and the second site was about 10 km downstream, just before the river flows into Lake Iskar. The Iskar River is the main source of water for nearly 80% of the households (approx. 1.3 million people) [17] and industrial needs of the capital city, Sofia, and Sofia region. As this is such a key freshwater ecosystem, we aimed to assess the impact of the WWTP effluent waters on the taxonomy, resistome and mobilome of the Iskar River. Samples were transferred to the laboratory within 6 h and processed immediately. A Lafil 400 vacuum filtration system (Rocker, Dist., New Taipei City 244014, Taiwan) was used with 0.2 µm 47 mm diameter nylon membrane filters (Cytiva, Whatman^tm,^, Little Chalfont, Buckinghamshire, UK) for filtration purposes. pH was recorded for each sample (Table S6). Each sample was homogenized, divided into two 500 mL portions and passed through two filters. Filters were stored at −80 °C until all samples were collected.

### 2.2. DNA Extraction and Sequencing

When the last pair of samples was acquired, all filters were aseptically cut into smaller pieces and subjected to DNA extraction in duplicate with the Environmental DNA & RNA Purification Kit (Cat. No. E3572, EURx, Gdańsk, Poland). Simultaneously, a Microbial Community Standard (ZymoBIOMICS, Zymo Research, Irvine, CA, USA, cat. D6300) was also subjected to DNA extraction and sequencing (Figure S4). DNA from replicates was measured both spectrophotometrically by BioDrop µLite+ (Biochrom Ltd., Harvard Bioscience, Inc. 84 October Hill Road Holliston, MA 01746, United States) and fluorometrically by Qubit 4.0 Fluorometer (Thermo Fisher Scientific, Waltham, MA, USA). The replicates with the highest 260/280 ratio and highest DNA concentration were used for NGS library construction with Collibri™ ES DNA Library Prep Kit (Thermo Fisher Scientific, USA) with 50 ng genomic DNA input. The libraries were pooled and sequenced on NextSeq 550 with V2.5 Mid Output kit (2 × 150 bp) (Illumina, San Diego, CA, USA). The sample information is provided in Table S1. 

### 2.3. Bioinformatic Analysis

Reads were automatically trimmed based on adapter content and average Q30+ quality score with the default setting of Trim-galore v0.6.7, ensuring that only high-quality reads were kept. Next, reads aligned to the human genome were removed with bmtagger and the remaining reads (from now on referred to as raw reads) were quality checked with FASTQC v0.11.9. 

For the purpose of co-assembly, three sets of reads were produced by concatenating all raw reads (C1-all), and reads per location (C2-Dragushinovo, C3-Mechkata). Co-assemblies were produced with megahit v1.2.9 [18] with default settings using kmers of length 21, 29, 39, 59, 79, 119, 141 and contigs shorter than 1000 bp were filtered out. Binning was performed on the 3 co-assemblies with MaxBin2 v2.2.7 [19], metaBAT2 [20], and CONCOCT v1.1.0 [21] separately by using the default parameters for each tool. Consolidation of the resulting metagenome-assembled genomes (MAGs) was performed by the bin refinement module of MetaWrap v1.3 [22]. Further, refined bins were subjected to the reassembly module of MetaWrap by first extracting reads mapping to the bins and feeding them to the SPAdes v.3.15.1 with different parameters. Afterwards, the resulting re-assemblies were compared and the best one by CheckM output were chosen as representative. Next, resulting MAGs were quality checked, annotated and uploaded to Bioproject PRJNA1071831 and were used for inference of host discovery of ARGs. All MAGs were quality checked with QUAST v5.2 and CheckM v1.2.2 [23]. 

Raw reads were used to identify ARGs with ResFinder software v2023-08-22 [24] and database v2023-04-12, applying 60% coverage and 60% identity thresholds. For ARGs from assemblies, both ResFinder and AMRFinderPlus v3.11.14 [25] were used. MGEs were searched within assemblies with MobileElementFinder v1.1.2 (https://pypi.org/project/MobileElementFinder/, accessed on 12 December 2023). 

Putative plasmids were identified using three plasmid prediction tools: Deeplasmid [26] with score above 0.75, PlaSquid v1.0.0 [27], and Plasme v1.1 [28]. The contigs identified as plasmids by at least one tool were included in the analysis. Co-occurrence of either ARG or MGE or both on contigs of plasmid origin were reported. MetaCompare2.0 was also used simultaneously for risk assessment of mobilizable ARGs on pathogens [29]. 

Taxonomic assignment to raw reads was performed with Kraken2 v2.1.2 [30] with the PlusPF (Standard plus Refeq protozoa & fungi), built in January 2023, followed by Bracken v2.8 [31] as previously described [32]. The resulting OTU count tables were imported into the Qiime2 platform [33], filtered from low count taxa (*n* = 20) as well as taxa found in less than 3 samples, and used for differential abundance analysis with the ANCOM-BC2 plugin [34]. Counts were transformed to relative frequencies, and taxa below 1% were merged into “Others” and used to visualize the taxonomic composition with matplotlib v3.7 and seaborn v0.12.0 Python packages. 

Alpha and beta diversity, group-significance, and volatility analyses were performed with ResistoXplorer [35] and Qiime2. Microbial community standard was analysed by submitting the raw reads to the miqScoreStandardShotgun tool v1.0.1. Network analysis was performed with the NetworkX Python package v3.2.1 by only including associations with positive Pearson correlation scores of ≥0.8 coupled with *p*-value ≤ 0.005. A network graph was then drawn in CytoScape 3.9.1. Statistical calculations were performed either automatically for ResistoXplorer and Qiime2 or by Scipy v1.11.1 for the network analysis. 

## 3. Results

Cleaned reads resulted in between 41.6–63.8 million reads of river samples and 2.20 million reads for the Mock Standard. Average read length per sample varied between 142 and 150 bp. Mock Standard results are shown in Figure S4.

### 3.1. Taxonomic Composition and Distribution

**Figure 1:**
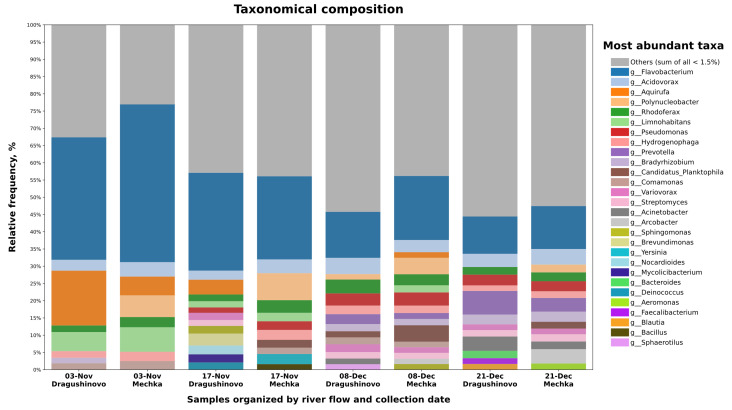
Taxonomic composition bar plot depicts the relative abundance of most abundant genera within each of the eight samples ordered by date and river flow. Low-abundant taxa (<0.1%) and taxa found in less than three samples were filtered out. Counts of level Genus were transformed into relative abundance and taxa below 1.5% were grouped into “Others”. The top 45 genera are designated, coded using distinct colours and ranked from most abundant to least abundant in descending order based on average relative abundance calculated across all samples.

Kraken2 followed by Bracken were utilized for the classification of raw reads into taxonomic ranks. For visualization purposes, only the Genus level was employed to depict the taxonomic composition (Figure 1). The full taxonomic table is available in Table S7. The principal phyla identified in the samples were predominantly *Pseudomonodota*, comprising 38% (at the early sampling dates) up to 60.7% (at the later dates), and *Bacteriodota*, exhibiting a gradual decrease from 54% to 21%. Within *Pseudomonodota*, *Betaproteobacteria* represented 28.6% at the class level, with *Burkholderiales* accounting for over 95% at order level. Among these, *Acidovorax*, *Polynucleobacter*, *Limnohabitans*, and *Rhodoferax* showed the highest relative frequencies and according to the literature were consistently found within the same habitat [36]. 

The most prevalent taxa across all samples included *Flavobacterium*, *Aquirufa*, *Acidovorax*, *Polynucleobacter* and *Limnohabitans* as determined by their average relative abundances. Additionally, genus *Flavobacterium* predominated within the phylum *Bacteriodota*, accounting for 70–73% during initial dates, decreasing to 60.6% by the third date, and further declining to 43.8% by the end of the study period. 

By observing the composition bar plot, clear trends become evident that some genera tend to decline with time and also there are distinct differences between each pair of samples by location. For instance, both *Flavobacterium* and *Aquirufa* diminished over the period of seven weeks, while others such as *Acidovorax* showed consistent levels across all samples. Furthermore, *Polynucleobacter* was significantly more abundant at the second location (villa area “Mechkata”). These trends seemed to be consistent across the four dates, suggesting that changes in the water composition from both locations are not due to random events and are not influenced entirely by the time span.

**Figure 2:**
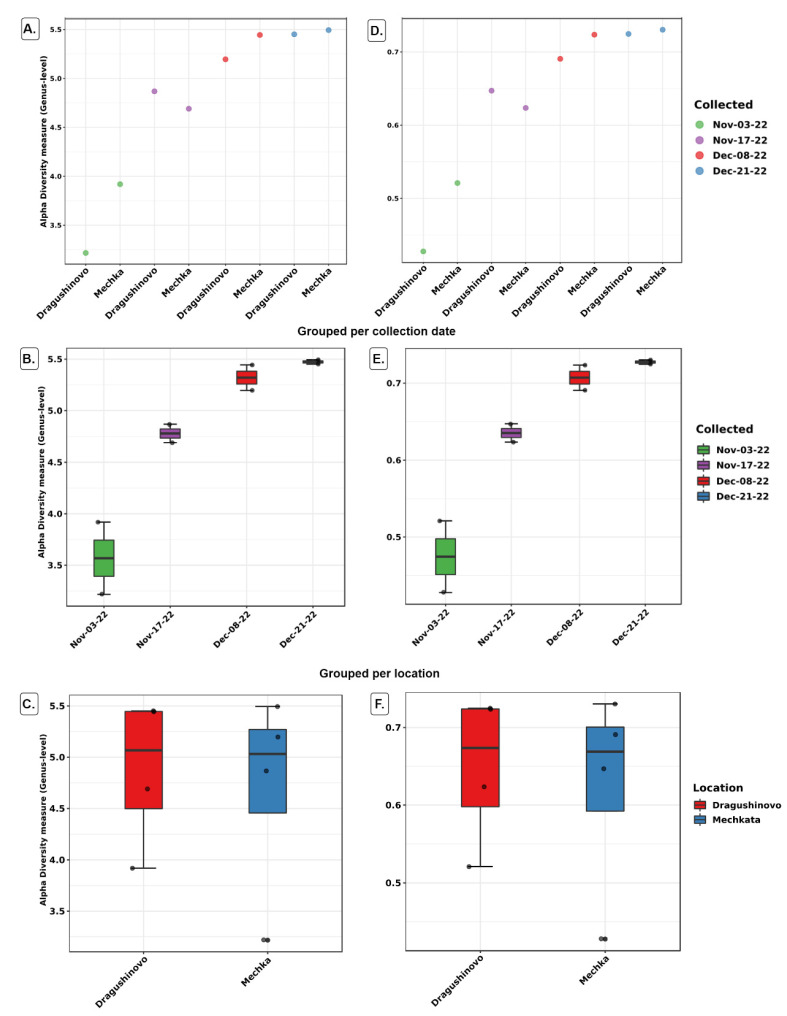
Alpha diversity and significance testing across samples by collection date and location. Shannon index measures overall diversity, incorporating both species richness (number of species) and evenness (distribution of species), whereas Pielou’s evenness specifically measures evenness. The graphs display on the y-axis the alpha diversity measures (Genus level) of Shannon (**A**–**C**) and Pielou’s evenness (**D**–**F**). (**A**,**D**) each sample individually; (**B**,**E**) in groups by collection date; (**C**,**F**) in groups by location. The spread and distribution of data points reveal the taxa richness and evenness increased over the time period (**B**,**E**) although not significantly (*p* = 0.08). No significant difference in Shannon and Pielou’s evenness diversity was observed between the two locations (**C**,**F**). Low count taxa (*n <* 5) were filtered out and 20% prevalence filter was applied, meaning that at least 20% of the values of a feature should contain at least five counts. Low variance filter based on standard deviation was also included. Data was normalized using Additive Log Ratio (ALR).

Shannon (Figure 2A–C) and Simpson (Figure 2D–F) alpha diversity metrics were used to compare samples either individually or in groups. Both indices showed an increase in the richness and the evenness in the species distribution over time. No difference was found between the two groups by location factor (Mann-Whitney = 8, *p* = 1). However, both indices showed a trend of convergence (not significant, Kruskal-Wallis = 6.6667, *p* = 0.08) between paired samples from the same date over the course of the study. Notably, the greatest disparity in indices was observed on the initial date, followed by a gradual reduction in differences on subsequent dates as also shown by the volatility graph (Figure S1). This was largely influenced by the decline of the genus *Flavobacterium*. The same analysis type was conducted with OTU tables of Family, Order and Class levels and the observations did not change.

### 3.2. Principal Coordinate Analysis (PCoA) and Differentially Abundant Analysis (DAA)

**Figure 3:**
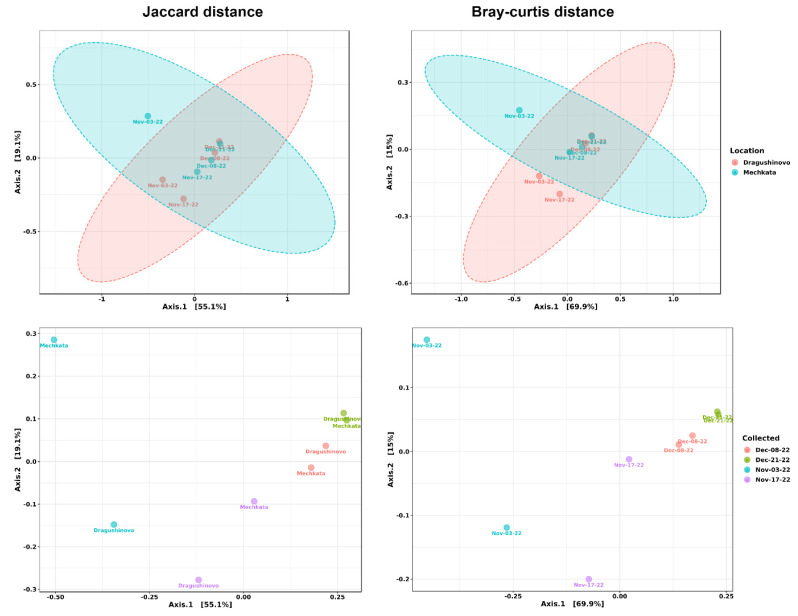
PERMANOVA tests applied to PCoA data using Bray-Curtis and Jaccard distance metrics to evaluate the differences in bacterial community composition between groups by addressing the abundance and presence/absence of species in each group. The results revealed no statistically significant differences between the groups, as demonstrated by the high F-values (0.68 and 0.72, respectively). The low R^2^ values further corroborated this observation, with the groupings accounting for only a small proportion of the overall variability in community composition. These findings indicate that the microbial communities are highly similar, regardless of the distance metric employed.

Next, PERMANOVA was used to determine if there were any significant differences in the composition of the bacterial communities between samples collected from different locations and dates by employing both Jaccard and Bray-Curtis distances to measure the dissimilarity between the circulating communities. The PCoA plots (Figure 3) revealed distinct separation between samples grouped by date (Bray-Curtis—F = 4.89, R^2^ = 0.79, *p* < 0.012; and Jaccard—F: 3.48, R^2^ = 0.72, *p* < 0.007), indicating that the taxonomic differences are more pronounced at each date as seen by the distinct separation of the pairs (Figure 3, bottom). In contrast, the overlapping clusters in the location-grouped PCoA plots (Figure 3, upper) indicated a high degree of similarity in microbial composition between samples taken from the same location (Bray-Curtis: F = 0.68, R^2^ = 0.10, *p* < 0.50; and Jaccard: F = 0.72, R^2^ = 0.10, *p* < 0.56). Group significance testing was also utilized to see if any significant changes occur between the two locations, and although the results are not significant (*p* = 0.576) the taxonomical variations in the first location (Dragushinovo village), which is immediately after the WWTP, are smaller compared to the downstream location (villa area “Mechkata”) (Figure S2), indicating that regardless of time, changes in bacterial composition are more pronounced at the second location. This trend could be due to a number of factors, including introduction of nutrients or substances such as antimicrobials by the WWTP effluent, temperature changes, flow patterns, and anthropogenic activities.

**Figure 4:**
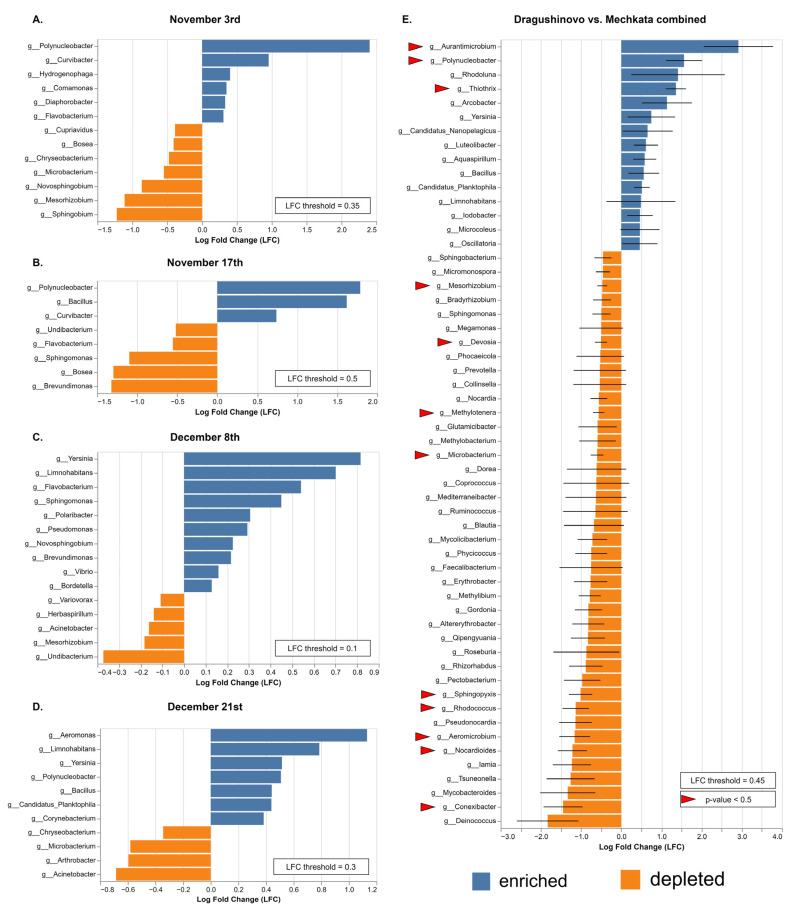
Differential abundance analysis (DAA) was utilized to identify enriched (blue) or depleted (orange) taxa when comparing samples both spatially and temporally based on their log-fold change (LFC) values. First, the OTU table was filtered to retain only taxa that were present in all eight samples and accounted for at least 0.1% of the total abundance across all samples. To identify which taxa tend to increase or decrease quantitatively, the ANCOM-BC plugin in Qiime2 was used to compare samples from Mechkata (downstream location) against Dragushinovo (upstream location, regarded as a reference) in each of the four collection dates: (**A**) 3 November; (**B**) 17 November; (**C**) 8 November; (**D**) 21 December; (**E**) all samples based on location. For E, less stringent filtering criteria have been applied, where taxa above 0.1% in at least two samples were included. This allowed genera not visible before, such as the genus *Aurantimicrobium*, to appear. Statistically significant (*p* < 0.5) results are indicated by red arrows. Different LFC threshold values were applied to all figures for visualization purposes. Full length figures are included in Figure S3.

While beta diversity analysis can identify overall patterns in bacterial community composition, DAA was applied to identify which taxa are driving these patterns between locations or time points, providing a more granular understanding of the underlying drivers of bacterial community composition changes (Figure 4). Genus *Polynucleobacter* ranked 4th in the overall abundance (Figure 1) but was assigned by ANCOMBC2 as significantly enriched (*p* < 0.5) in the downstream location in all four dates. *Polynucleobacter* has a much higher Log-fold change (LFC) in the early dates (1.75–2.4) compared to the last two dates (0.4–0.5), which could be attributed to temperature variations and corresponded with previous studies on temporal changes in taxonomy of freshwater ecosystems [37,38]. *Aurantimicrobium* was present above 0.1% in 7/8 samples and was significantly increased (*p* < 0.5) in the second location. Genus *Curvibacter* was enriched over the course of the riverflow in the first two sampling dates. There was a shift of differentially abundant bacteria between early and late observation dates. In the last two dates, genera such as *Yersinia* (0.5–0.8 LFC, *p* < 0.5), *Limnohabitans* (0.7–0.8 LFC, *p* < 0.5), *Flavobacterium* (0.1–0.53 LFC, *p* > 0.5), *Vibrio* (0.09–0.15 LFC, *p* > 0.5) and *Bordetella* (0.04–0.13 LFC, *p* > 0.5) were enriched.

### 3.3. ARGs and ARG-Host Association

**Figure 5:**
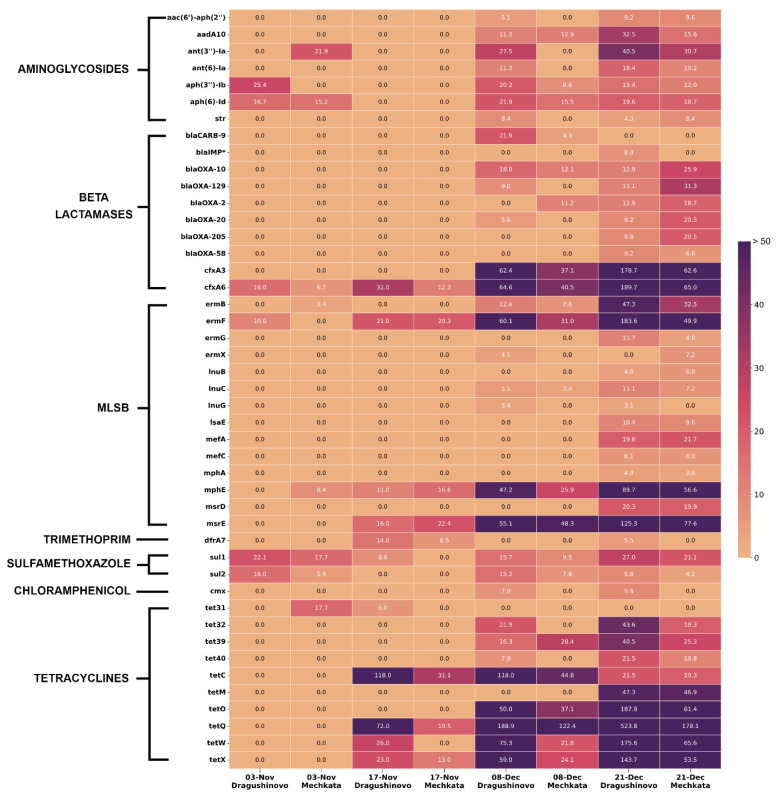
A heatmap graph showing normalized reads mapping to ARGs of clinical importance. ARGs found in fewer than two samples were excluded except for the IMP gene, which is marked with *. Found genes with a minimum of 60% coverage and 60% identity were included. Genes were ordered based on the class of antimicrobials they confer resistance to. Due to the high number of reads mapping to certain genes in comparison to low-represented ones, the colour thresholds were manually adjusted. All genes with *n* = 50+ reads were coloured the same. MLSB refers to ARGs that affect varying combinations of: erythromycin, azithromycin, telithromycin, lincosamide, streptogramin B/A, lincomycin, clindamycin, quinupristin, and dalfopristine.

**Figure 6:**
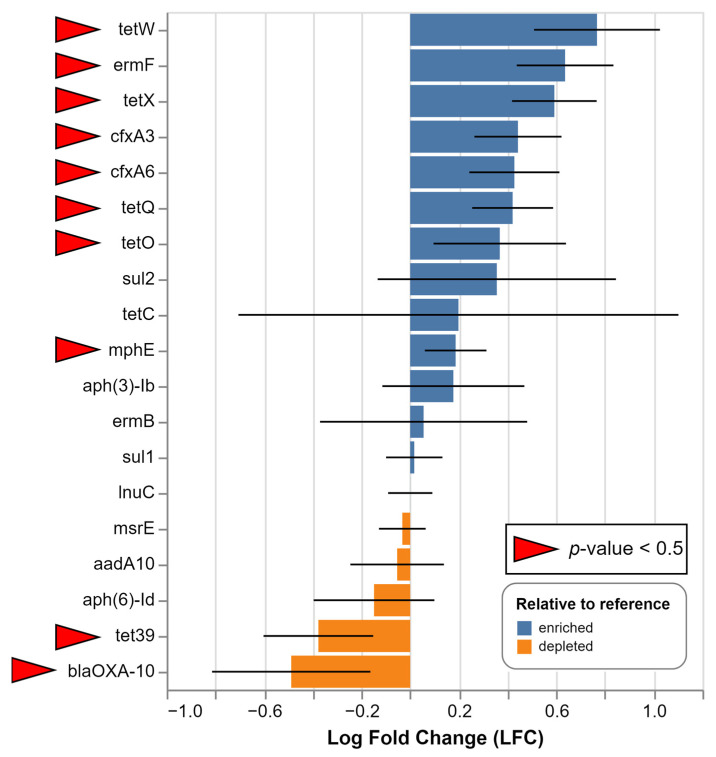
Differential abundance analysis (DAA) was utilized to identify enriched (blue) or depleted (orange) ARG based on their log-fold change (LFC) values between Dragushinovo (closer to the WWTP) and Mechkata locations. Only ARG found in at least four samples were included. The ANCOM-BC plugin in Qiime2 was used to compare samples from Mechkata (downstream location, regarded as a reference) against Dragushinovo (upstream location) in all dates together. Statistically significant (*p* < 0.5) results are indicated by red arrows.

We identified clinically important ARGs that lead to reduced antimicrobial susceptibility and summarized them in Figure 5. Among them, the most abundant were the groups conferring resistance to macrolides and tetracyclines, followed by the beta-lactams, aminoglycosides (mainly streptomycin), and sulfonamides. There was a clear trend of increased ARG abundance in the samples from the last two dates and in particular at Dragushinovo located directly downstream of the WWTP. A number of ARGs were found to be significantly enriched at the Dragushinovo site (Figure 6).

This result could not be attributed to differences in sequencing depth, due to the comparable number of reads obtained from all samples and the complete absence of some ARGs in the first four samples. Also, the same analysis was repeated with subsamples of random reads from the latest four samples and the heatmap pattern remained similar. Interestingly, we detected two carbapenemases—*bla*OXA-58 and *bla*IMP-like. *bla*OXA-58 was found with 97.51% identity and coverage and also identified within the C2-Dragushinovo co-assembly with 100% match. In regard to *bla*IMP, a few reads from S7-Dragushinovo partially mapped with the IMP-13 gene (77.46% ID and coverage), whereas the *bla*IMP gene with 100% coverage and 95.5% ID to *bla*IMP-33 appeared within the C1-all co-assembly. Additional research is needed to establish the exact variant, and there is likelihood of the presence of multiple IMP variants based on the aligned reads to the IMP-13 and IMP-33 sequences. Nonetheless, the sheer presence of both genes within the freshwater Iskar River is alarming as they are usually detected in hospital settings and occasionally in animal farms.

**Figure 7:**
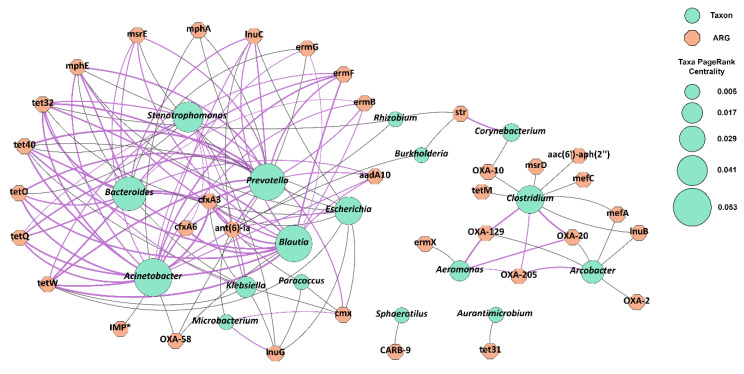
Bipartite network graph showing host-ARG associations. Only the 40 most highly represented taxa and additional known human pathogens were included. ARGs found in fewer than two samples were excluded except for the IMP gene, which is marked with *. Green circular nodes represent the genera, whereas orange octagons are ARGs. Edges link nodes from both groups based on positive Pearson correlation (≥0.8) and *p*-value (≤0.005), and their colour and thickness denote the weight of the connection (purple is higher). Positive correlations indicate a high theoretical probability of a taxon acting as a host for an ARG. Node sizes of genera denote the centrality of each node to the network. The visual length of the edges should be ignored, as the graph layout was manually adjusted to enhance readability. Please note that not all edges in the network may represent meaningful relationships.

For the purposes of finding a putative host of all the clinically relevant ARGs found by ResFinder, we extracted the number of reads mapping with a high degree of certainty and calculated Pearson correlation between them and the taxa found in the samples (Figure 7). Two clusters formed out, with one of them incorporating numerous ARGs for tetracycline (*tetQ*, *tetW*, *tetO*, *tet40*, *tet32*), macrolide (*ermB*, *ermF*, *ermG*, *mphE*, *msrE*, *mph*), and beta-lactamases genes and the key taxa *Acinetobacter* (18 edges), *Bacteroides* (15 edges), *Prevotella* (17 edges), *Blautia* (17 edges), *Stenothrophomonas* (12 edges), *Escherichia* (10 edges), and *Klebsiella* (9 edges). The statistically significant enrichment of ARGs such as *tetQ*, *tetW*, *tetO*, *ermF*, *mphE*, *cfxA3*, *cfxA6*, and *ermB* at the first location, Dragushinovo (near the WWTP discharge), is shown in Figure 5 and Figure 6. These ARGs were positively correlated with genera such as *Prevotella*, *Blautia*, *Bacteroides*, *Escherichia*, *Klebsiella*, *Acinetobacter*, and *Clostridioides* (Figure 7), which were also enriched at the first location (Figure S3 and Table S7). Notably, many of these taxa are either opportunistic human pathogens or native to human microbiomes, not native water microbiota. Additionally, *Prevotella* and *Blautia* are indicators of human faecal contamination in water [39]. The depletion of both the ARGs and these taxa towards the downstream location, coupled with their significant correlation, led us to believe that they were not prevalent in the water at baseline and must be introduced by the WWTP.

### 3.4. Co-Occurrence of ARG and MGE on Putative Plasmid Sequences and Resistome Risk Score

To identify putative plasmids, we subjected the co-assemblies (C1-all, C2-Dragushinovo, and C3-Mechkata) to three different plasmid prediction tools (Section 3.2). The results from the tools yielded varying levels of agreement, with some contigs identified as plasmids by multiple tools, whereas the majority were identified by only a single tool. To capture a comprehensive view of potential plasmids, we included all contigs flagged as plasmids by at least one tool. As a result, in the assemblies C2 and C3, 3386 and 1897 contigs were flagged as plasmid-borne, respectively. They were extracted, grouped and submitted for ARG and MGE detection. Co-occurrences of ARGs and MGEs on plasmids were rare and three instances were identified in total: *bla*OXA-58 and ISAlw25 found in C2-Dragushinovo only; tn917 and *erm(B)* in C2 only; and Tn6082 carrying *aph(6)-Id* and *aph(3″)-Ib* in C2 and C3. Interestingly, more putative plasmids and more instances of co-occurrences were found in the WWTP effluent waters at the first location, although the assemblies had comparable N50 (2159 bp vs. 2196 bp) and total length (621 M bp vs. 475 M bp).

*The bla*OXA-58 carbapenemase and ISAlw25 were located on a 1256 bp contig with 34 bp gap in between, which in turn was identified by both PlaSquid and PLASMe. The most closely related plasmid was pOXA58_005069 (NZ_CP026086.2) from *Acinetobacter pitti*, whereas according to ISFinder ISAlw25 was found in *A. lwoffii* plasmid. Essentially, *bla*OXA-58 reads were detected only in the last sampling date (Figure 5). However, a fully reconstructed gene with 100% coverage and ID was achieved only in C1-all and C2-Dragushinovo co-assemblies and not from C3-Mechkata, indicating the possible higher abundance of OXA-58 in the first location compared to the downstream location. Tn917 (Tn551 in TnCentral) carrying *ermB* was located on a 5717 bp contig identified by PlaSquid and PLASMe. The macrolide resistance *ermB* gene is a native element of Tn917 and is also widely distributed in *Streptococci*, *Enterococci*, and *Bacteroides* [40,41], which aligned well with the host-ARG association analysis of *ermB* with *Bacteroides*, *Prevotella*, *Acinetobacter* and *Blautia* (Figure 7). Tn6082 carrying *aph(6)-Id* and *aph(3″)-Ib* is very abundant in clinical strains of *Enterobacterales* and *Pseudomonas*. It was found in ESBL-producing *E. coli* isolates from pigs in South Africa [42] and in *Klebsiella* isolates from freshwater lakes in India [43]. 

Plasmids are also regarded as MGEs and even though the co-occurrence of MGEs and ARGs on plasmids may be limited due to factors such as incomplete assembly or sequencing depth, it is still important to note the presence of the remaining ARGs identified on putative plasmid sequences alone due to their potential for HGT. Of note, in both locations the following additional genes were detected: *aph(2″)-If* (amikacin, gentamicin, tobramycin, kanamycin); *blaOXA-1* (ESBL), *blaOXA-10* (ESBL, aztreonam), and *blaOXA-20 (penicillins)*; *sul1* and *sul2* (sulfamethoxazole); *tet(39)*, *tet(C)*, *tet(O)*, *tet(W)* and *tet(X2)* together conferring resistance to doxycycline, tetracycline, minocycline, and tigecycline; *erm(B)*, *mph(E)*, *msr(E)* all together conferring resistance to erythromycin, azithromycin, clindamycin, among others. The last three were found located on a single contig (in C2-Dragushinovo) with high assembly coverage compared to other ARGs, highlighting their abundance and putative co-transfer.

MetaCompare 2.0 pipeline was used to calculate the resistome risk score for both locations and for the river overall. Assemblies from individual samples were of low sequencing depth probably due to the high bacterial heterogeneity, therefore co-assemblies C1, C2, and C3 were assessed separately (Table S4). The combined resistome risk factor from all eight samples assessed by submitting the C1 co-assembly was perceived as a representative score for the sampling location of the river over the study period and is estimated to be 6.28 for ecological niches and 0.19 for human health, which is considered of low risk according to the authors of MetaCompare. This was in accordance to the low number of found co-occurrences of ARGs and MGEs. The risk scores by location were highly similar to the combined scores.

### 3.5. Recovery of MAGs

In total, 29 metagenome-assembled genomes were produced from the C1-all co-assembly. Bins with ≥70% completion and ≤10% contamination were produced from each binning tool, then refined by the MetaWrap bin refinement tool and reassembled with Spades with varying sets of parameters to produce improved assembly with higher N50 and completion to contamination ratio. Next, the MAGs were quality-assessed by Busco and CheckM and submitted to NCBI (PRJNA1071831). Of all MAGs, five were high-quality MAGs (≥90% completion and ≤5% contamination) and the rest were medium quality (MiMAGs according to [44]). The GTDB-TK tool with GTDB database Release 214 were used to identify each MAG and assign the lowest taxonomic rank. Detailed information on resulting MAGs is available in (Table S5). We also reconstructed MAGs from the other two co-assemblies, C2-Dragushinovo and C3-Mechkata, however the number of MAGs in each was lower than in C1-all. In general, recovered MAGs originated from the most abundant genera (Figure 1) and the number of recovered MAGs increased with sequencing depth as seen in the current analysis. MAG bin.2.orig classified to genus JAAFJM01 (*Bacteroidota bacterium*) carried aminoglycoside modifying aph(3*″*)-Ib and aph(6)-Id. The high-quality MAG *s_Agathobacter rectalis* (bin.12.permissive) was identified to carry *ermG*, and the bacterium is natively found in the rumen of cow and sheep, indicating a potential animal faecal contamination. In addition, g_*Aurantimicrobium* (bin.19.orig) carried a *tet* gene with high identity to *tet(37).*

## 4. Discussion

There are a few approaches to determine the microbial communities and/or the presence of ARGs in water bodies such as targeted PCR detection, 16S, or shotgun metagenomics [45]. Recent advances in sequencing technologies have enabled and transformed shotgun metagenomics into a much sought-after and affordable technique for such purposes and thus revolutionized our ability to study the microbial communities that inhabit diverse environments. This is the first comprehensive shotgun metagenomic study focused on documenting the bacterial taxonomic diversity of a freshwater river in Bulgaria immediately following a WWTP, extracting and constructing MAGs, describing taxonomic temporal and spatial variations, and elucidating the abundance of ARGs, with a particular emphasis on those of clinical relevance. At the time of writing, in Bulgaria there was only one published study from 2017 on 16S rRNA metagenomic profiling of the bacterial freshwater communities in two Bulgarian reservoirs [46], showing the vastly unexplored area. In regard to AMR, a few attempts to detect AMR in water bodies could be listed; however, all of them were culture-based studies [47,48,49,50], therefore with limited potential for a more detailed description of both the microbial communities and their ARGs. Lastly, one molecular-based targeted detection study was published on the freshwater Iskar Dam that is located downstream of our sampling locations; however, the study focus was water-borne pathogens with emphasis on nontuberculose mycobacteria [51]. 

The full potential of high-throughput sequencing data is often not realized, as many studies focus on a limited subset of analyses, such as the detection and analysis of ARGs. While ARG profiling and detection of anthropogenic factors such as intl1 [52,53,54] and ARGs of clinical relevance such as carbapenem hydrolysing variants of OXA, KPC, and NDM is essential for understanding the spread of AMR, they represent only a fraction of the information that can be derived from sequencing data. Our study extended beyond that by integrating metagenome-assembled genomes (MAGs) into our interpretation of the ARGs, by revealing their antimicrobial resistance genetic potential, thus complementing ARG discovery. In addition, we have also applied the MetaCompare2.0 risk assessment calculation to provide another perspective, thereby expanding the understanding of AMR in the environment and encouraging the further development and standardization of such risk assessments to provide a quantitative metric to facilitate future decision making by stakeholders.

In general, the bacterial composition in all samples corresponded well with the taxonomical structure observed in both lentic and lotic freshwater habitats [37,55,56,57,58,59]. The two genera *Prevotella* and *Blautia* were found among the top 30 most represented genera in the samples. Both were found to decrease (not significantly) in all pairs of samples between the two locations and also positively correlated to the same 17 clinically important ARGs for macrolides and tetracyclines, and were also suggested to be predictors for human faecal contamination due to their presence in the gut microbiome [39]. Other studies also found ARGs such as cfxA3, tetQ, and ermF in *Prevotella* [60,61,62] with tetQ often located on a conjugative transposon or together with ermF [62,63]. Also, the Samokov WWTP lacks tertiary treatment, that is proven to further reduce bacterial load in folds in the effluent water [64,65]. The network analysis also identified other taxa that include opportunistic pathogens such as *Acinetobacter*, *Klebsiella*, *Escherichia* and *Clostridioides*, potentially serving as hosts for a number of clinically relevant ARGs, and considered to originate from the WWTP as an anthropogenic contamination.

In our study, the ARGs found in the water were highly congruent with results from other studies on freshwater aquatic systems: ARGs conferring aminoglycoside resistance such as aminoglycoside phosphotransferases *(3*′*)-la*, *(3*′*)-lb*, and *(6)-ld* [66,67,68]; nucleotidyltransferase commonly found in WWTPs sludge *aadA*, *aacA4*, *aadB*, *aadE*, *strB* [68,69]*;* ARGs conferring resistance to macrolides such as *ermB* [70,71]; *msrE* and *mphE* [72]*; sul1* and *sul2* [71,72,73,74]; and *tet* genes [70,71,72,73,74,75]. The most disturbing was the clear presence of *bla*OXA-58 and *bla*IMP-33-like carbapenemase genes. *bla*OXA-58 is frequently found in *Acinetobacter* isolates in clinical settings and was the most prevalent in several countries in Europe [76,77]. Most importantly, it confers resistance to the last-line antimicrobials imipenem and meropenem. The enzyme was also prevalent in water from coastal areas and was associated with a variety of host bacteria [78]. Additionally, it was found in higher absolute abundance in effluent WWTPs water compared to upstream locations or downstream locations [53,54,60,61,62,63,64,65,66,67,68,69,70,71,72,73,74,75,76,77,78,79], which aligned well with the current results. In this regard, the comparison of acquired resistance and co-occurrence of ARGs and MGEs identified more hits in the first location, Dragushinovo, that is immediately after the WWTP, thus being consistent with the results of other studies [66,67,68,69,70,71,72,73]. These studies also described an elevation of the abundance of resistome and mobilome of the WWTP effluent waters compared to either upstream or further downstream sites. 

A 2019 study reported a negative association between the abundance of ARGs in WWTP effluents and the number of biological treatment steps employed in the WWTP [53]. The Samokov WWTP was built in 2001 and at that time it was the most advanced WWTP in the Balkans with mechanical, biological, and sludge management but lacking tertiary treatment. Although the plant covers a region of 125,000 equivalent number of inhabitants and is the largest WWTP operated by the “Sofiyska Voda”, it remains the oldest one without upgrades to date. As such, the Samokov WWTP would have to be included in regular surveillance of AMR on wastewaters and WWTPs, as is evidenced by the proposed directive of the European Commission in the context of “One Health” [80] and as strongly recommended by other authors [81,82]. According to authors, it is now clear that human activities shape the levels and types of AMR encountered in freshwater ecosystems [8]. However, there are still critical questions that must be addressed such as what kinds and levels of ARGs pose a risk of acquiring a resistant infection and what other environmental factors, such as concentrations of antimicrobials or heavy metals, could be the selective pressure for resistant microbes and ARGs. Therefore, in future studies we plan to include assessment of the residual levels of antimicrobials in the water.

A few limitations of the study should be noted. Samples from the first location are not directly representatives of the WWTP effluent waters. The first location, Dragushinovo, is approximately 600–800 m downstream of the actual WWTP location, and as a result the precise impact of the WWTP effluent waters on the mobilome and the resistome of the river could not be assessed. Our future studies could potentially be improved by increasing the sampling sites and specifying more critical sampling points such as upstream sites or WWTP influent water to more precisely describe the impact of WWTP on the resistome of the river. In this regard, the initial study plans were to assess the impact of the numerous sheep, cattle, goat and cow farms in the surrounding river region by hypothesizing an overall increase in the ARGs between the two sampling locations. The reason this particular location was chosen was due to dense population with livestock farms and the fact that the region (South-West) is also the second largest breeding/producing in the country, as shown in the 2022 country report on Livestock Farms [83]. In addition, to date there is an overuse of antimicrobials in the veterinary sector coupled with loose regulatory practices from the government as documented in a 2019 European Commission report on antimicrobial use in Bulgaria [84]. Therefore, we assumed that farms coupled with the imprudent use of antimicrobials act as a source of ARG selection and dissemination into the groundwaters and the Iskar River. However, the results of the survey suggest the opposite. A decrease in ARGs with river flow can be explained by the high levels of water dilution. Nonetheless, dairy cattle faecal bacteria such as *Agathobacter rectalis* validates the contaminating impact of animal breeding to the freshwater quality. Next, we used a microbial community standard to be able to measure the extent of the bias introduced by DNA extraction and library preparation. While the MIQ score tool yielded a good score of 67 (Figure S4) and all the included bacteria were retrieved and identified, there is still room for pre-sequencing protocols optimization.

## 5. Conclusions

This is the first study providing valuable insights into the bacterial diversity and antimicrobial resistance profiles within the River Iskar, a major freshwater drinking source of great economic importance. The identification of both faecal contaminants and acquired ARGs, including primarily healthcare-associated carbapenemase genes such as *bla*OXA-58 and *bla*IMP, that should not occur naturally in the environment, highlighted the impact of anthropogenic contamination on freshwater ecosystems. The results indicate that clinically important ARGs and some opportunistic pathogens at the point of water discharge are most likely introduced by the WWTP Samokov. These data demonstrate the possibility of uncontrolled spread of AMR beyond the healthcare sector and into the environment. Moreover, since some of the ARGs were detected on plasmids or within transposons, there are certain risks associated with further spread of AMR into non-resistant bacteria through horizontal gene transfer, which in turn may act as a reservoir for AMR or pose a direct threat to human health. The findings are crucial to inform strategies to mitigate the environmental spread of AMR such as comprehensive monitoring and systematic reporting of quantitative data, and improving outdated WWTPs. 

## Data Availability

All data employed are included in the main text and in the Supplementary Materials. Sequencing data and resulting Metagenome-assembled genomes are available under the Bioproject PRJNA1071831. Raw data are available here: SRP487318. MAGs biosamples are available from SAMN39991762 through SAMN39991790. Relevant links and/or references to other sources are included in the main text. The generated information and/or datasets analysed during the current study are available from the corresponding author on reasonable request.

## Supplementary Materials

The following supporting information can be downloaded at: https://www.mdpi.com/article/10.3390/microorganisms12061250/s1, Figure S1: Dynamics of alpha diversity indices; Figure S2: Beta diversity group significance; Figure S3. Differential abundance analysis (DAA) in full length; Figure S4: MIQ shotgun sequencing report on Microbial Test Standard; Figures S5 and S6: Bubbleplot with antimicrobial resistance genes; Table S1: Cleaned reads; Table S2: Assemblies per sample Quest; Table S3 Co-assemblies Quast; Table S4: MetaCompare2.0; Table S5: MAGs; Table S6: Sample pH; Table S7: Taxonomic composition.
