# Supplementary material for: Metagenomic Investigation of the Short-Term Temporal and Spatial Dynamics of the Bacterial Microbiome and the Resistome Downstream of a Wastewater Treatment Plant in the Iskar River in Bulgaria
Source: Microorganisms. 2024 Jun 20;12(6):1250. doi: 10.3390/microorganisms12061250 (PMC11207046; doi:10.3390/microorganisms12061250)
Supplement: Supplementary file 1 [file microorganisms-12-01250-s001.zip › suppl-combined-rIskar-final.pdf]

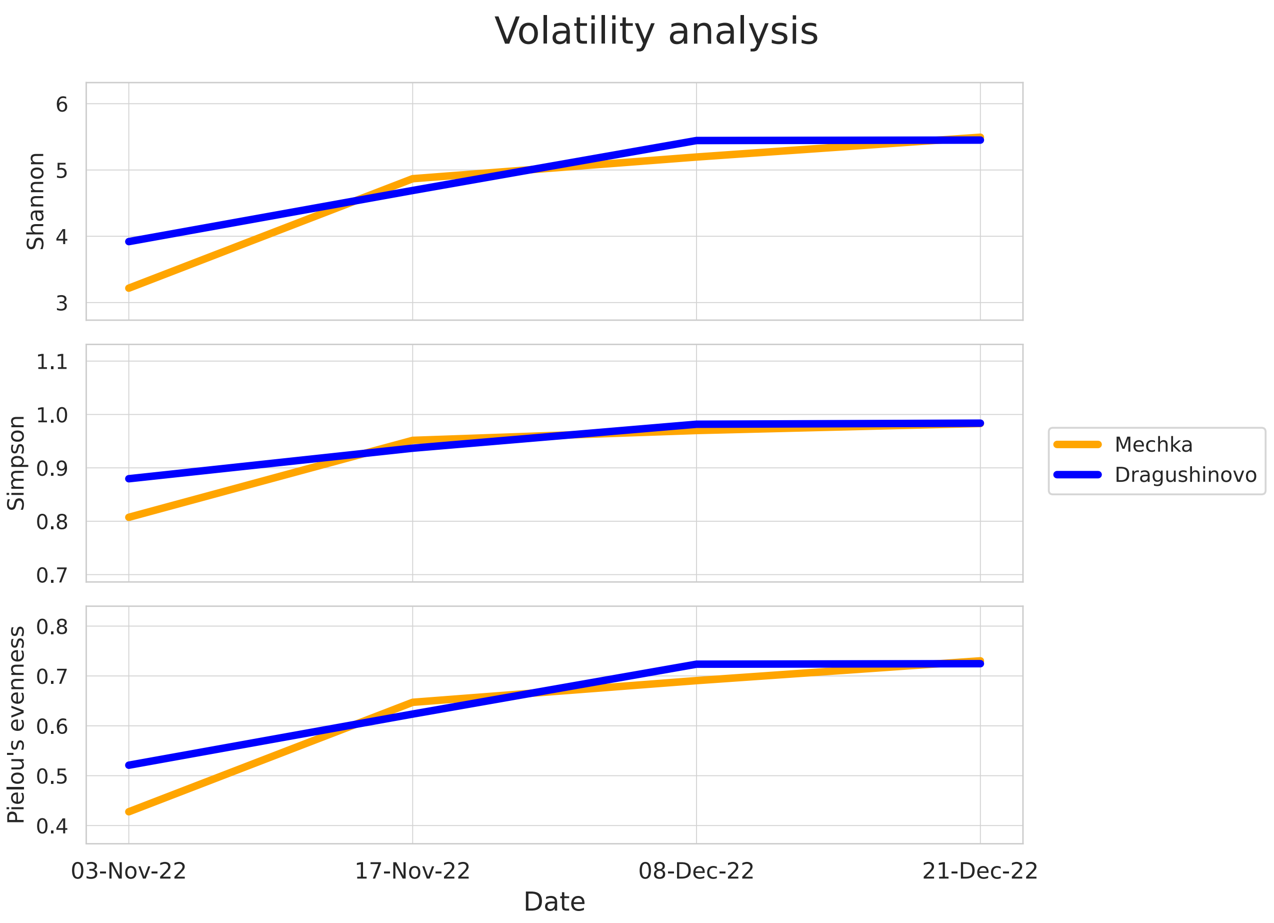

Supplementary Figure S1. Dynamics of alpha diversity indices. This figure illustrates the temporal dynamics of alpha diversity metrics (Shannon, Simpson and Pielou's evenness) across eight samples collected from two distinct locations "Dragushinovo" (blue line) and "Mechkata" (orange line) over the course of four sampling dates. The line plots depict variations in the diversity metrices, revealing richness and evenness change over time. The volatility analysis highlights the increase in taxonomic richness in both locations as time progressed towards the last sampling date. Low count taxa (*n* < *5*) were filtered out of the OTU table and also 20% prevalence filter meaning that at least 20% of the values of a feature should contain at least 5 counts. Low variance filter based on standard deviation was also included. Datawas normalized using Additive Log Ratio (ALR).

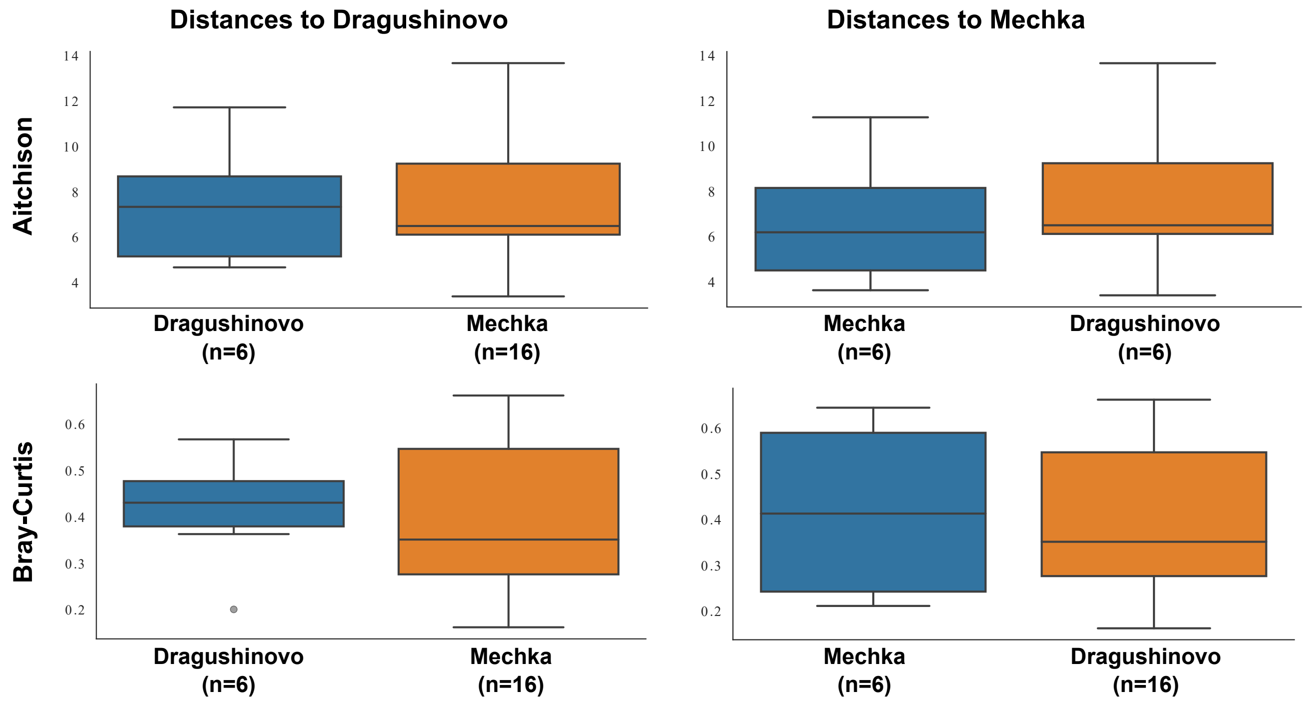

Supplementary Figure S2. Beta diversity group significance. The Bray-Curtis and Jaccard distance metrics ware utilized to calculate the pairwise dissimilarities within and between locations, to reveal the extent in the variation in microbial community. The analysis was created within Qiime2 platform.The figure displays the clustering of the differences of beta diversity indices between each pair of samples used to assess microbial community dissimilarities between the two sample locations (Dragushinovo and Mechka). Taxonomic diversity at Dragushinovo location displayed less variation compared to Mechka, although not significantly ($p < 0.05$).

November 3rd

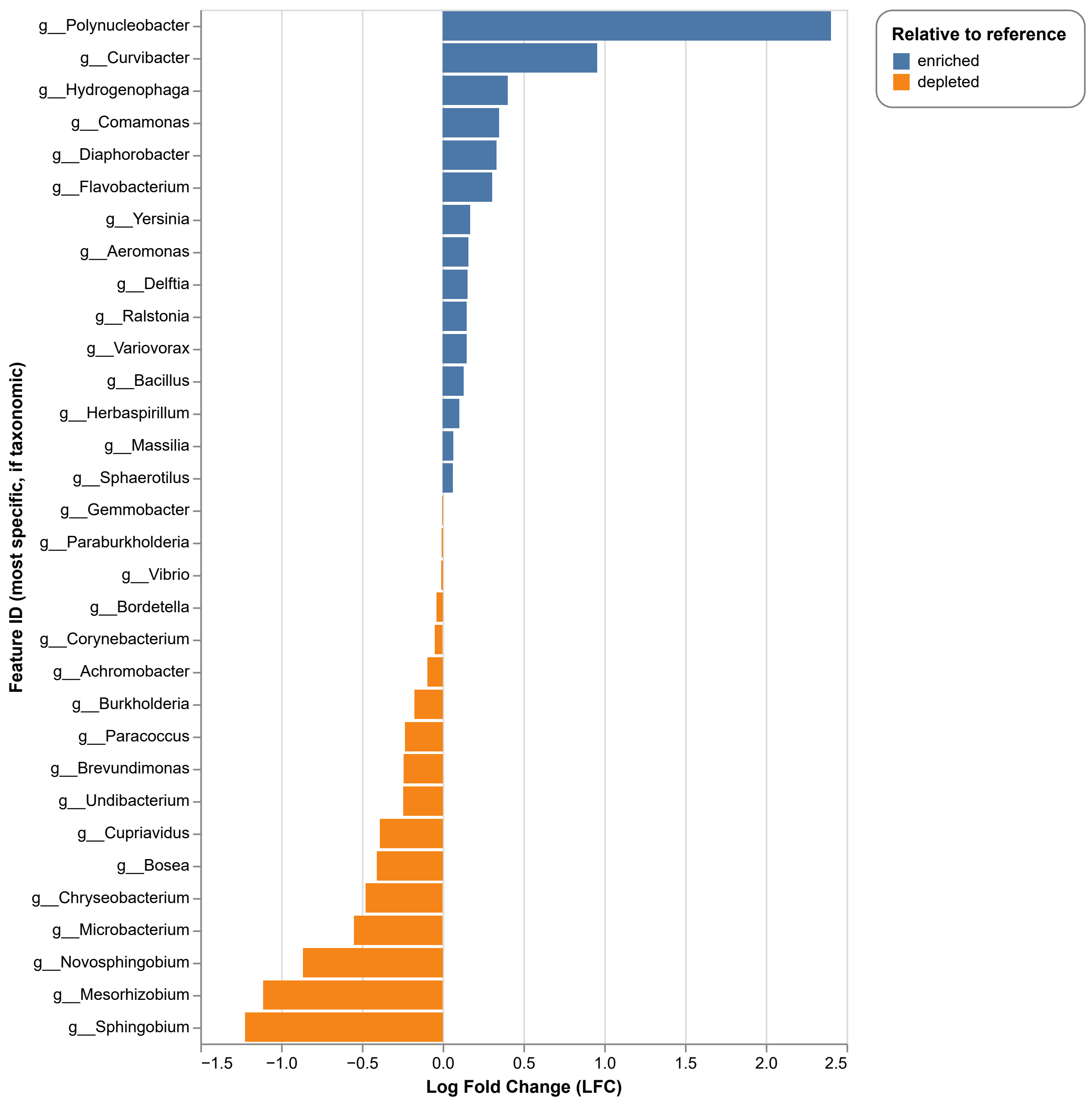

November 17th

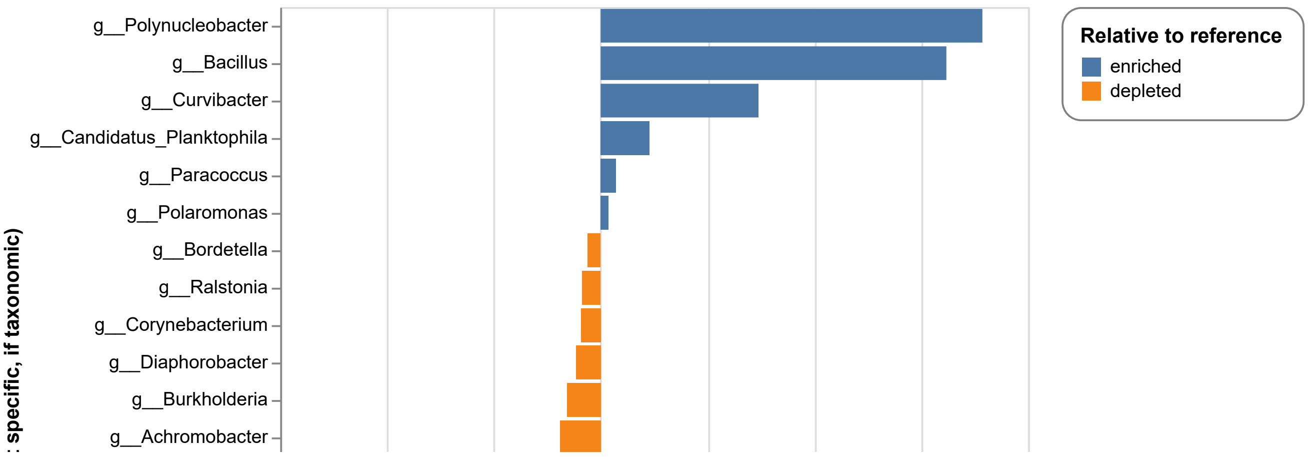

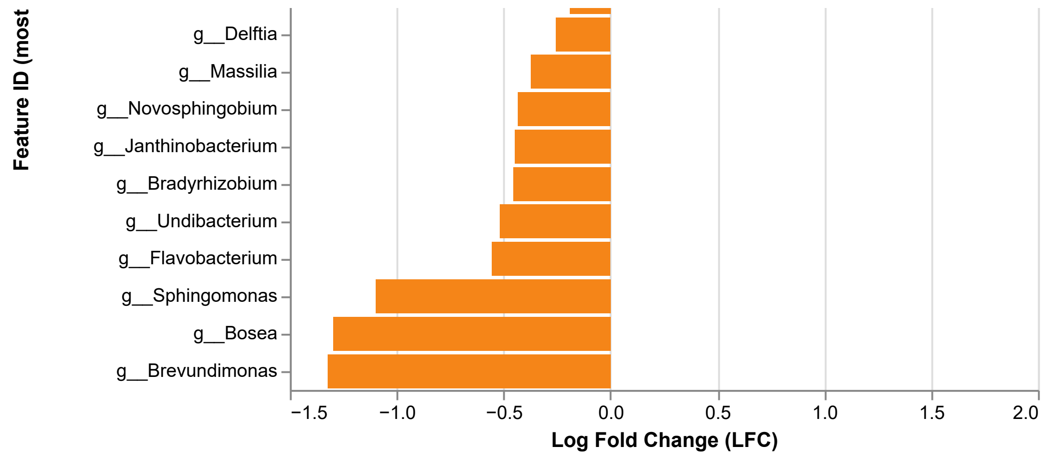

December 8th

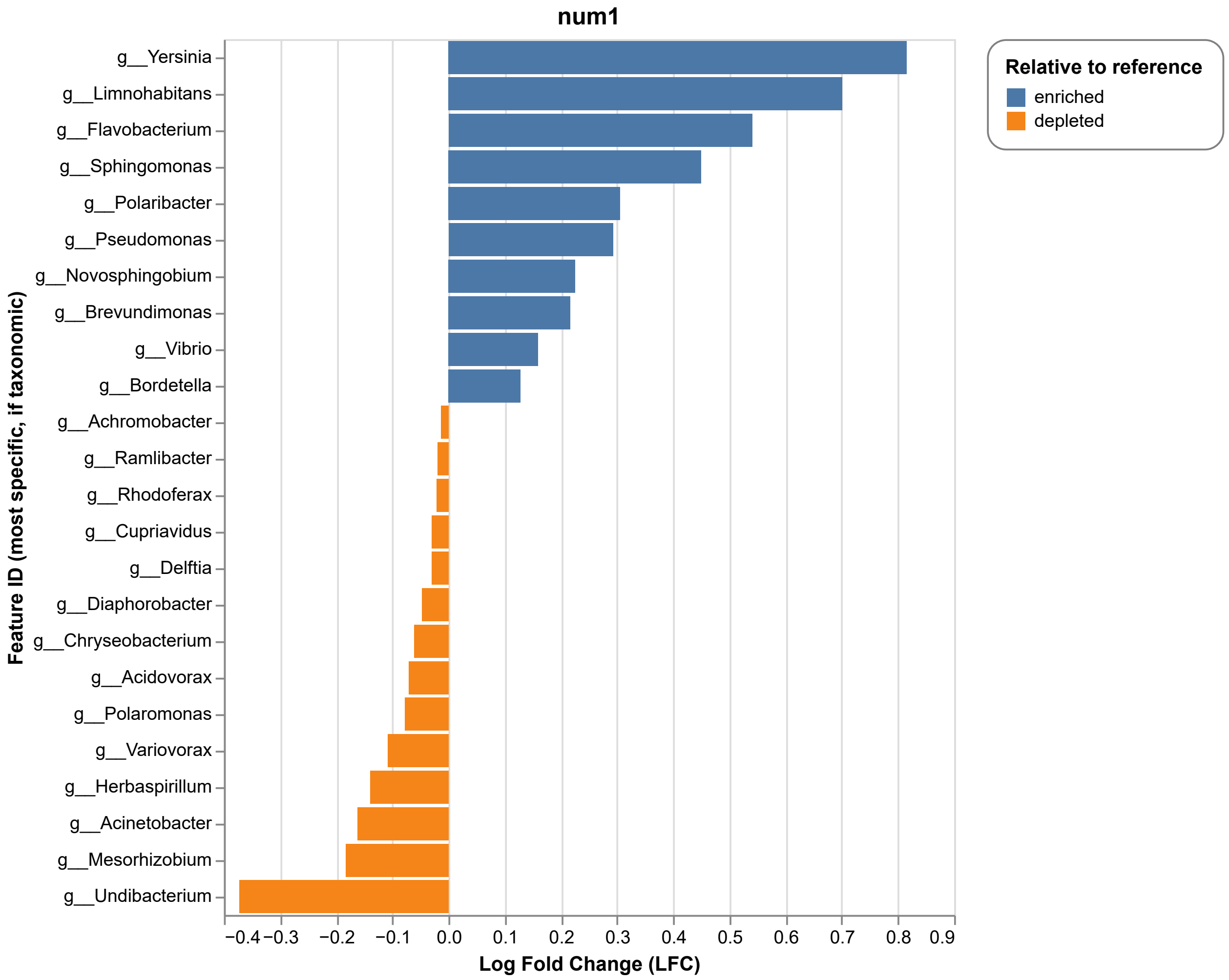

December 21st

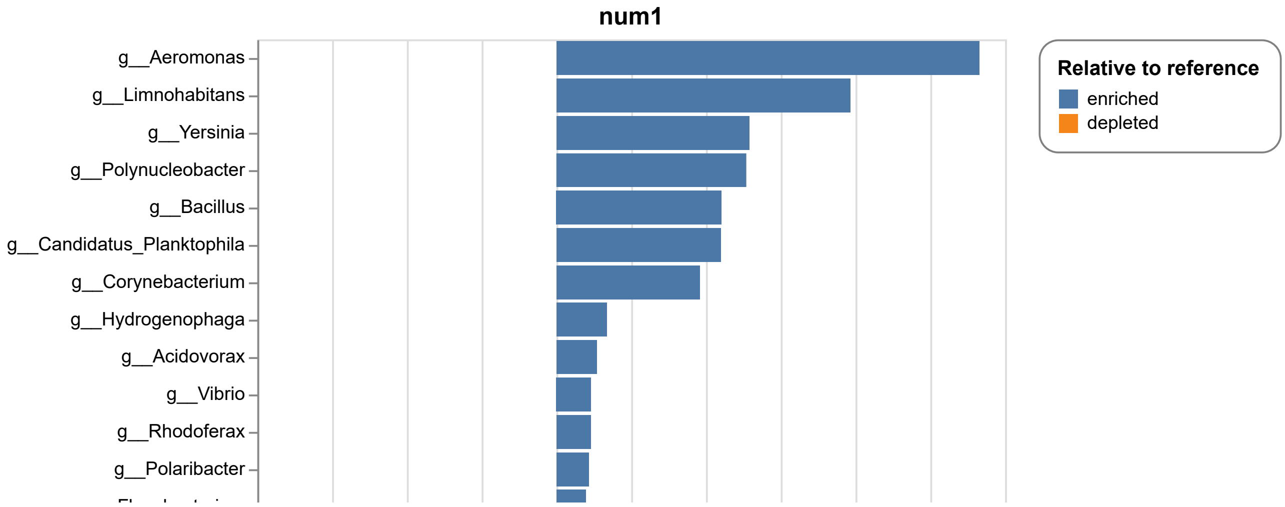

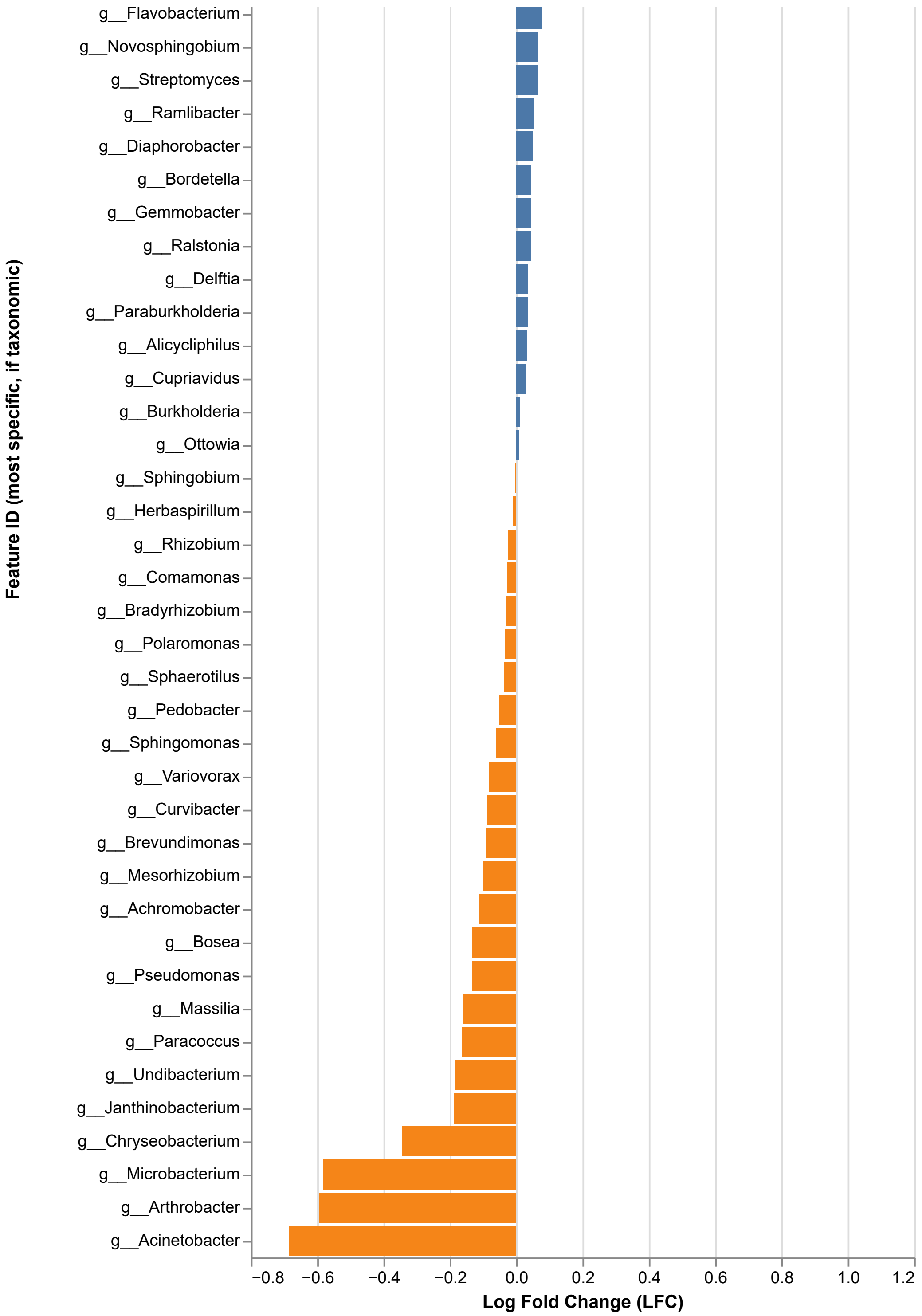

Dragushinovo vs. Mechkata combined

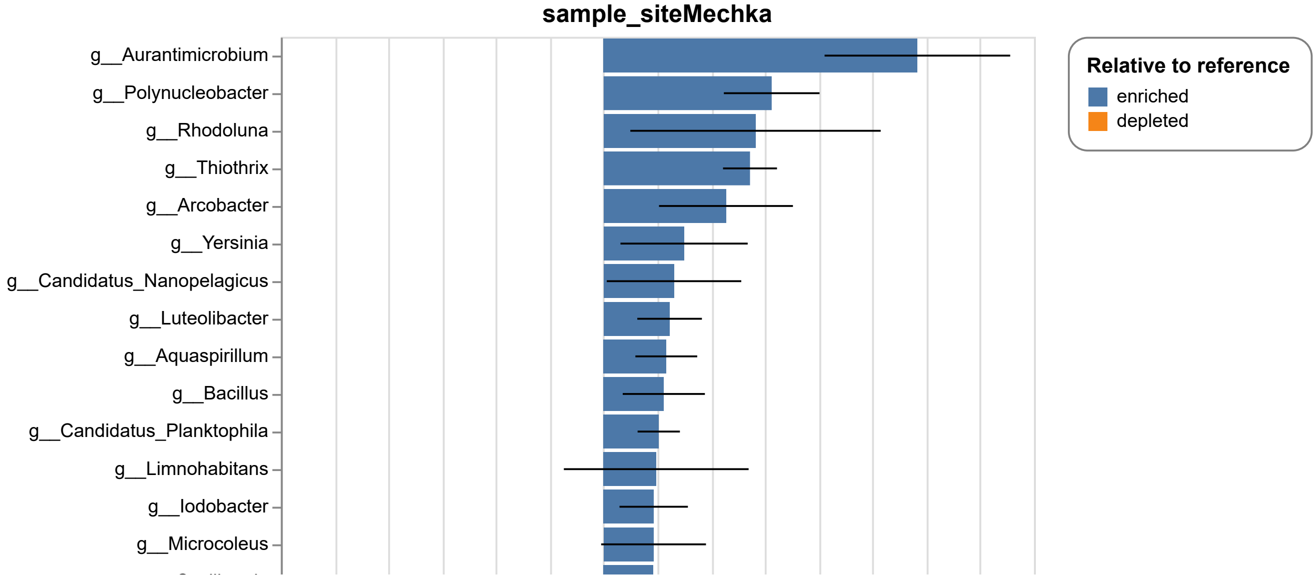

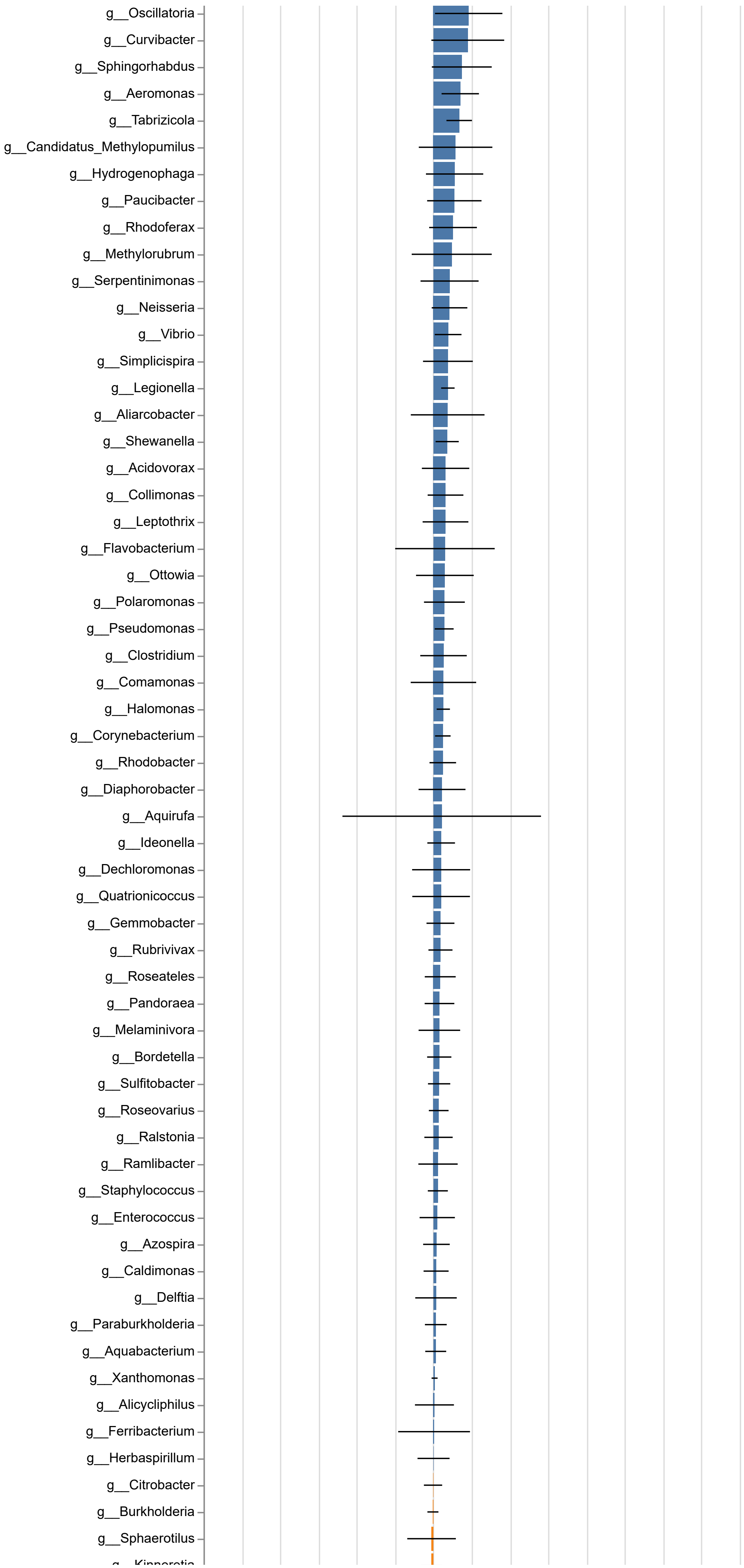
g__Oscillatoria
g__Curvibacter
g__Sphingorhabdus
g__Aeromonas
g__Tabrizicola
g__Candidatus_Methylopumilus
g__Hydrogenophaga
g__Paucibacter
g__Rhodoferax
g__Methylorubrum
g__Serpentinimonas
g__Neisseria
g__Vibrio
g__Simplicispira
g__Legionella
g__Aliarcobacter
g__Shewanella
g__Acidovorax
g__Collimonas
g__Leptothrix
g__Flavobacterium
g__Ottowia
g__Polaromonas
g__Pseudomonas
g__Clostridium
g__Comamonas
g__Halomonas
g__Corynebacterium
g__Rhodobacter
g__Diaphorobacter
g__Aquirufa
g__Ideonella
g__Dechloromonas
g__Quatrionicoccus
g__Gemmobacter
g__Rubrivivax
g__Roseateles
g__Pandoraea
g__Melaminivora
g__Bordetella
g__Sulfitobacter
g__Roseovarius
g__Ralstonia
g__Ramlibacter
g__Staphylococcus
g__Enterococcus
g__Azospira
g__Caldimonas
g__Delftia
g__Paraburkholderia
g__Aquabacterium
g__Xanthomonas
g__Alicycliphilus
g__Ferribacterium
g__Herbaspirillum
g__Citrobacter
g__Burkholderia
g__Sphaerotilus

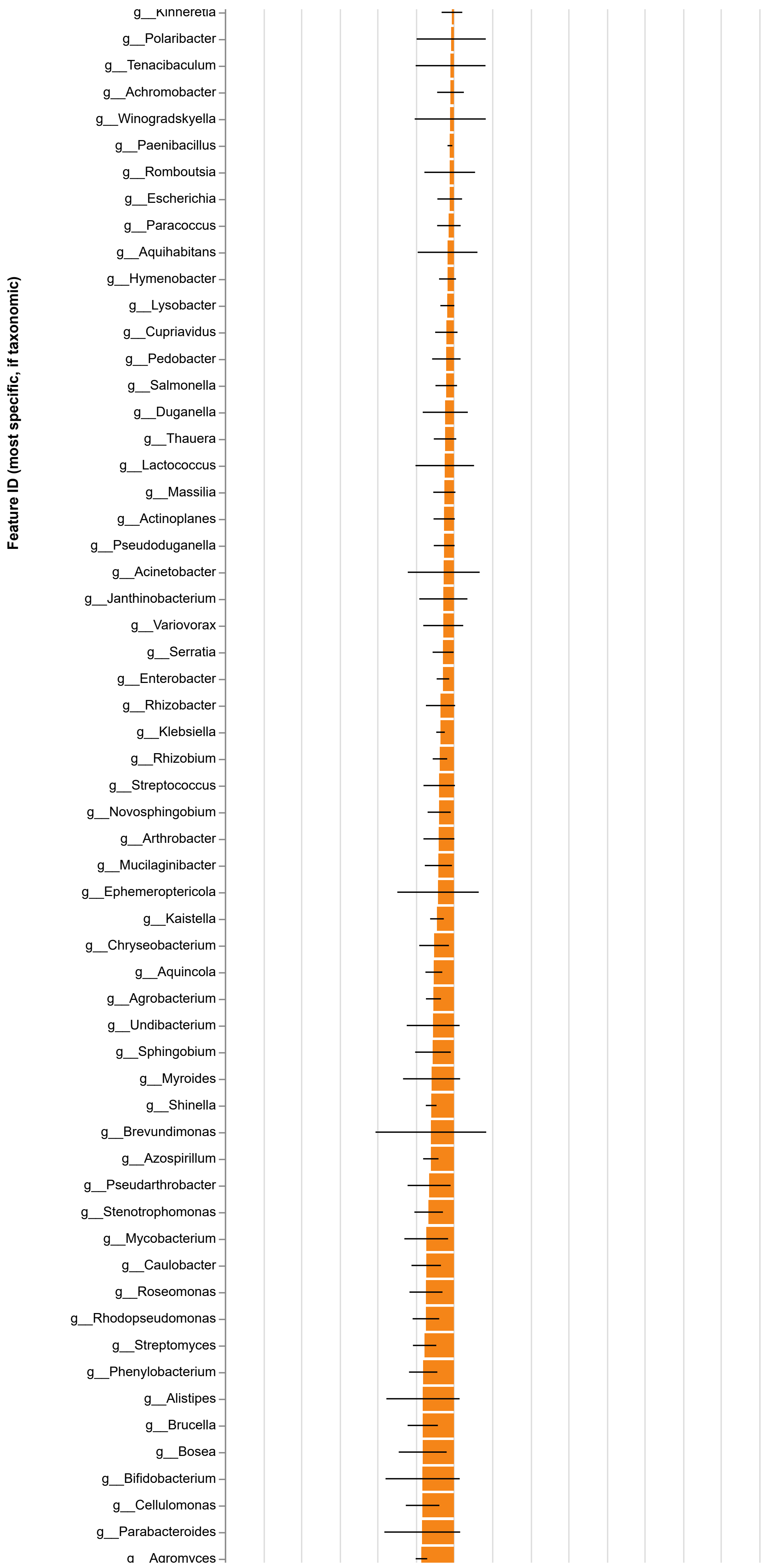

Feature ID (most specific, if taxonomic)
g__Kinneretia
g__Polaribacter
g__Tenacibaculum
g__Achromobacter
g__Winogradskyella
g__Paenibacillus
g__Romboutsia
g__Escherichia
g__Paracoccus
g__Aquihabitans
g__Hymenobacter
g__Lysobacter
g__Cupriavidus
g__Pedobacter
g__Salmonella
g__Duganella
g__Thauera
g__Lactococcus
g__Massilia
g__Actinoplanes
g__Pseudoduganella
g__Acinetobacter
g__Janthinobacterium
g__Variovorax
g__Serratia
g__Enterobacter
g__Rhizobacter
g__Klebsiella
g__Rhizobium
g__Streptococcus
g__Novosphingobium
g__Arthrobacter
g__Mucilaginibacter
g__Ephemeroptericola
g__Kaistella
g__Chryseobacterium
g__Aquincola
g__Agrobacterium
g__Undibacterium
g__Sphingobium
g__Myroides
g__Shinella
g__Brevundimonas
g__Azospirillum
g__Pseudarthrobacter
g__Stenotrophomonas
g__Mycobacterium
g__Caulobacter
g__Roseomonas
g__Rhodopseudomonas
g__Streptomyces
g__Phenylobacterium
g__Alistipes
g__Brucella
g__Bosea
g__Bifidobacterium
g__Cellulomonas
g__Parabacteroides
g__Agromyces

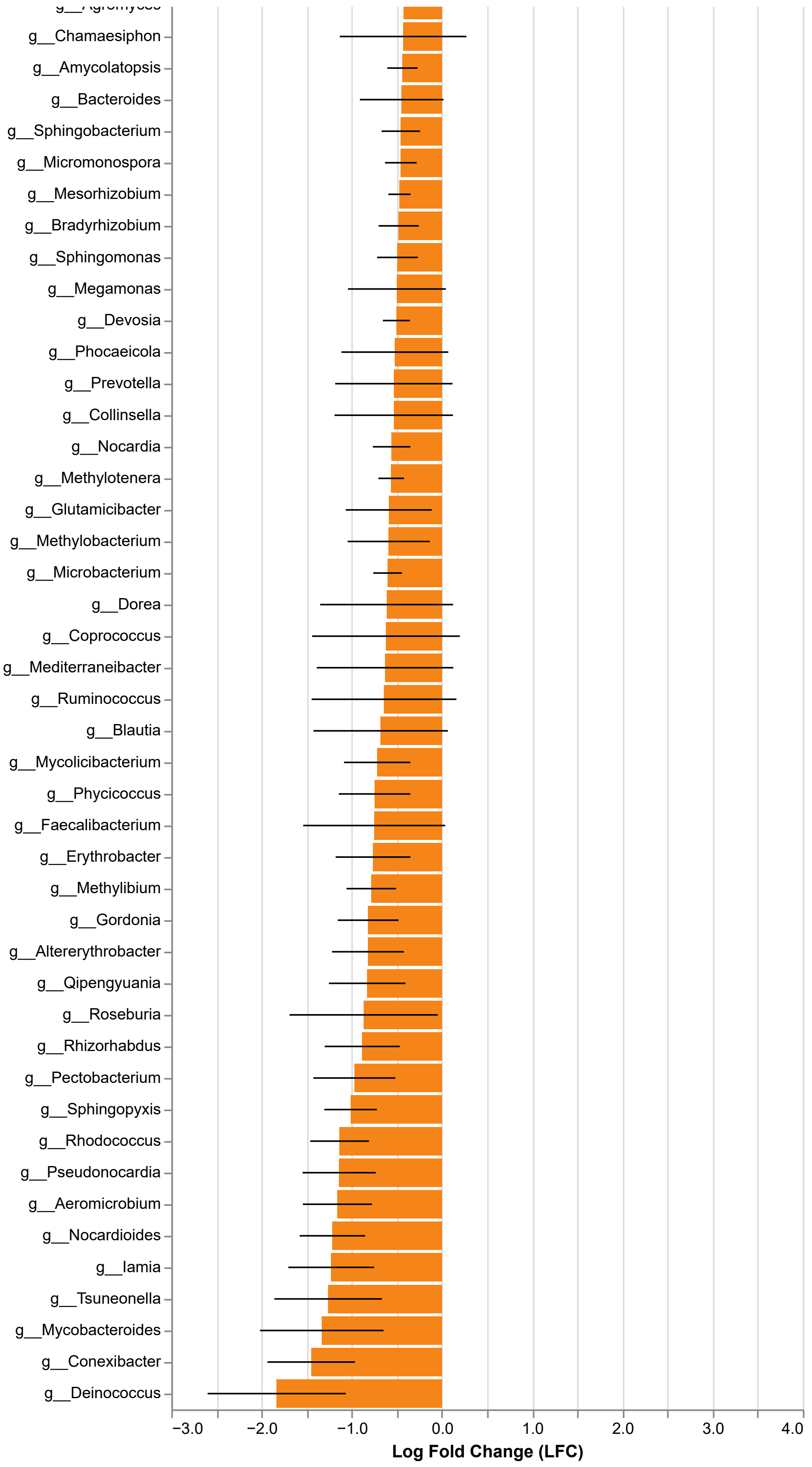

Supplementary Figure S3. Differential abundance analysis (DAA) in full length. Enriched (blue) or depleted (orange) taxa when comparing samples both spatially and temporally based on their log-fold change (LFC) value. First, OTU table was filtered to retain only taxa that were present in all eight samples and accounted for equal or above 0.1% of the total abundance across all samples. To identify which taxa tend to increase or decrease quantitatively ANCOM-BC plugin in Qiime2 was used to compare samples from Mechkata (downstream location) against Dragushinovo (upstream location, regarded as a reference) in each of the four collection dates. From top to bottom are 3rd of November; 17th of November; 8th of November;21st of December; All samples based on location. Error bars are shown as black lines.

MIQ SHOTGUN SEQUENCING REPORT

Sample ID: Iskar_river_mock

1. **MIQ (Measurement Integrity Quotient Score)**

1. MIQ (Measurement Integrity Quotient Score)

Your score: **67**

The miq score is used with a mock community or other known input standard with known manufacturing tolerances. The formula for calculating this score is based off of the root mean squared of errors with an adjustment for known variability in the standard itself. This number is a single metric that represents the entire complex pipeline. As such, a low score can be due to multiple factors. The bias identification (radar) plots below can suggest specific biases in analysis (such as bias against hard-to-lyse organisms).

2. **Bias Detection (Radar) Plots**

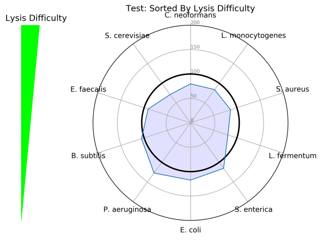

Good (Example)

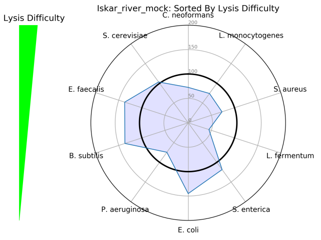

Iskar_river_mock

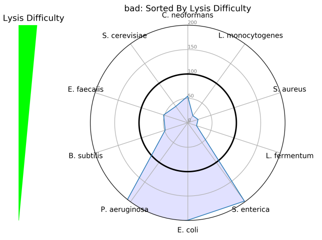

Biased (Example)

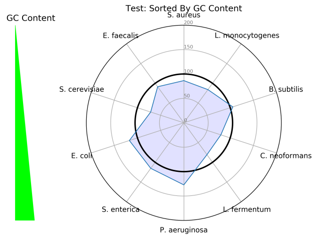

Good (Example)

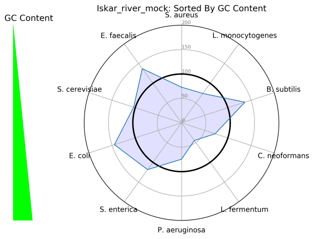

Iskar_river_mock

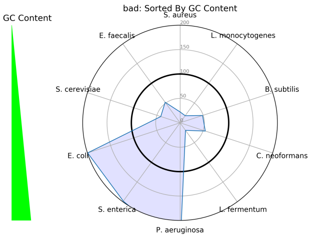

Biased (Example)

Radar plots, while they ultimately show similar data to the composition bar plots, are extremely useful because they can show the observed proportion of each organism relative to the expected, and organisms with similar features or behaviors can be grouped together opposite those with differing properties. An ideal plot should have all the points and mass collected around the 100% mark, indicating that the observed proportion of each organism was at or near 100% of expected. In general, if the mass appears to be shifted up or down, there was likely to have been a bias with regard to that property. Additionally, if the mass appears to be flattened from the top and bottom and widened in the central area, that may represent a bias against both extremes.

3. **Sample Composition**

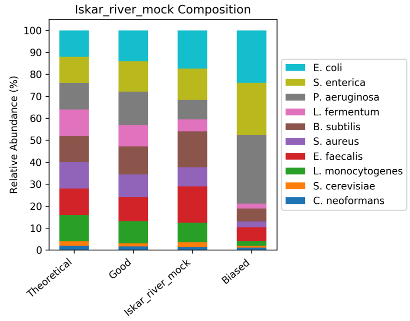

4. **Read Fate Counts**

Read Fate	Count
Poor_quality	29173
Chimera_like	1062
Unaligned_reads	3662
Aligned To Reference	1078679

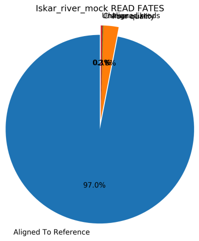

Read fates can be used to determine many major issues with your sequencing run. Ideally, nearly every read should map to the expected reference genome for the standard. These values are best compared to other standard analyses run under the same conditions. A significant change in the total number of reads could indicate a possible failure of the sequencing run or library prep, as would a larger than expected proportion of reads being filtered out for poor quality. Large numbers of reads that are of good quality, but cannot be mapped to the reference genome for the standard, might suggest a potential contaminant in the sample. Finally, every analysis method can have its own specific read filtering behaviors, such as chimeric read removal in 16S ribosomal RNA gene analysis.

Supplementary Figure S4. MIQ shotgun sequencing report on Microbial Test Standard. The formula for calculating this score is based off of the root mean squared of errors with an adjustment for known variability (≤ 15%) in the standard itself. Ideally, every read should map to the expected reference genome for the standard, which would equal MIQ score of 100. There are no published thresholds for consistent interpretation, but higher score means less bias introduced.

Total

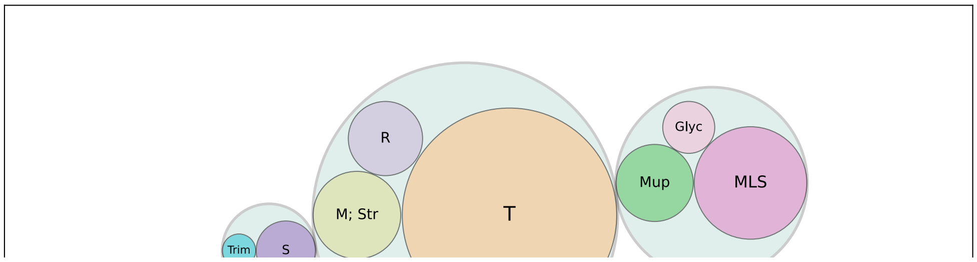

Drug classes abbreviations

B-pen - penam

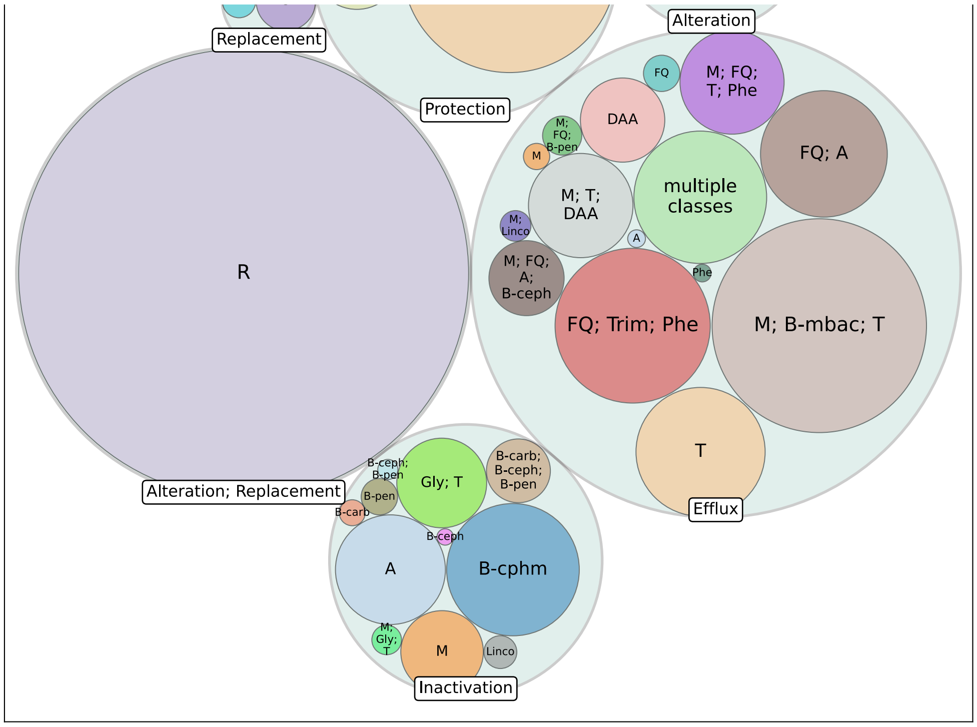

B-cphm - cephamycin
B-mbac - monobactam
B-ceph - cephalosporin
B-carb - carbapenem
T - tetracycline antibiotic
FQ - fluoroquinolone antibiotic
A - aminoglycoside antibiotic
M - macrolide antibiotic
MLS - macrolide antibiotic; lincosamide antibiotic; Str; streptogramin A antibiotic; streptogramin B antibiotic
Glyc - glycopeptide antibiotic
Linco - lincosamide antibiotic
Gly - glycylcycline
Trim - diaminopyrimidine antibiotic
S - sulfonamide antibiotic
R - rifamycin antibiotic
Oxaz - oxazolidinone antibiotic
Phe - phenolic antibiotic
Fos - phosphonic acid antibiotic
Pep - peptide antibiotic
Mup - mupirocin-like antibiotic
Nitr - nitrofuran antibiotic
DAA - disinfecting agents and antiseptics
multiple classes - more than 4 classes

Supplementary Figure S5. Bubbleplot with antimicrobial resistance genes extracted from raw reads from CARD DB v3.2.7 with 60% coverage and 60% ID. Genes were grouped by drug class for general representation. Results from CARD DB were not considered and included in the Result section of the manuscript due to the high number of reported intrinsic genes.

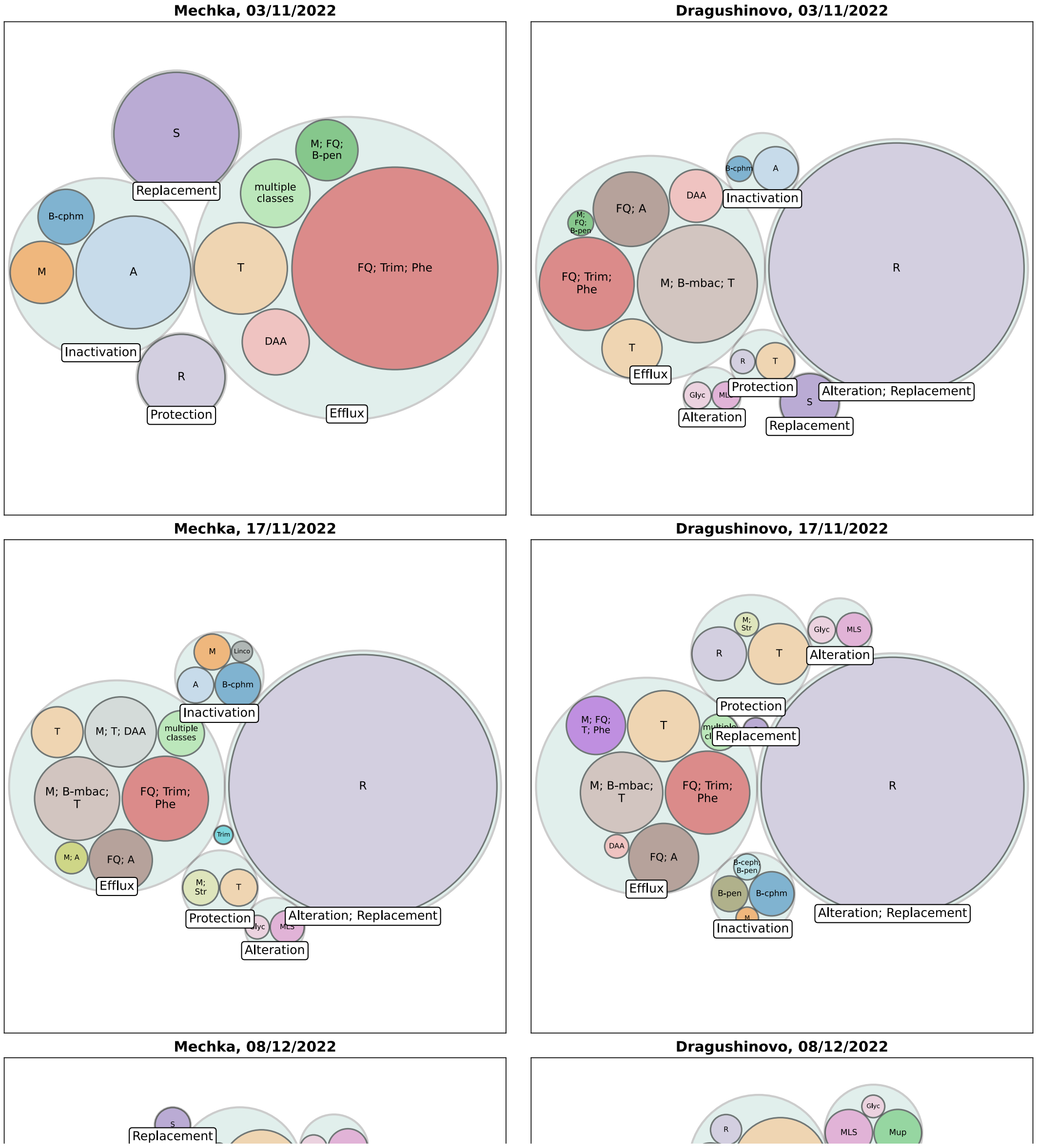

Drug classes abbreviations

B-pen - penam
B-cphm - cephamycin
B-mbac - monobactam
B-ceph - cephalosporin
B-carb - carbapenem
T - tetracycline antibiotic
FQ - fluoroquinolone antibiotic
A - aminoglycoside antibiotic
M - macrolide antibiotic
MLS - macrolide antibiotic; lincosamide antibiotic; Str; streptogramin A antibiotic; streptogramin B antibiotic
Glyc - glycopeptide antibiotic
Linco - lincosamide antibiotic

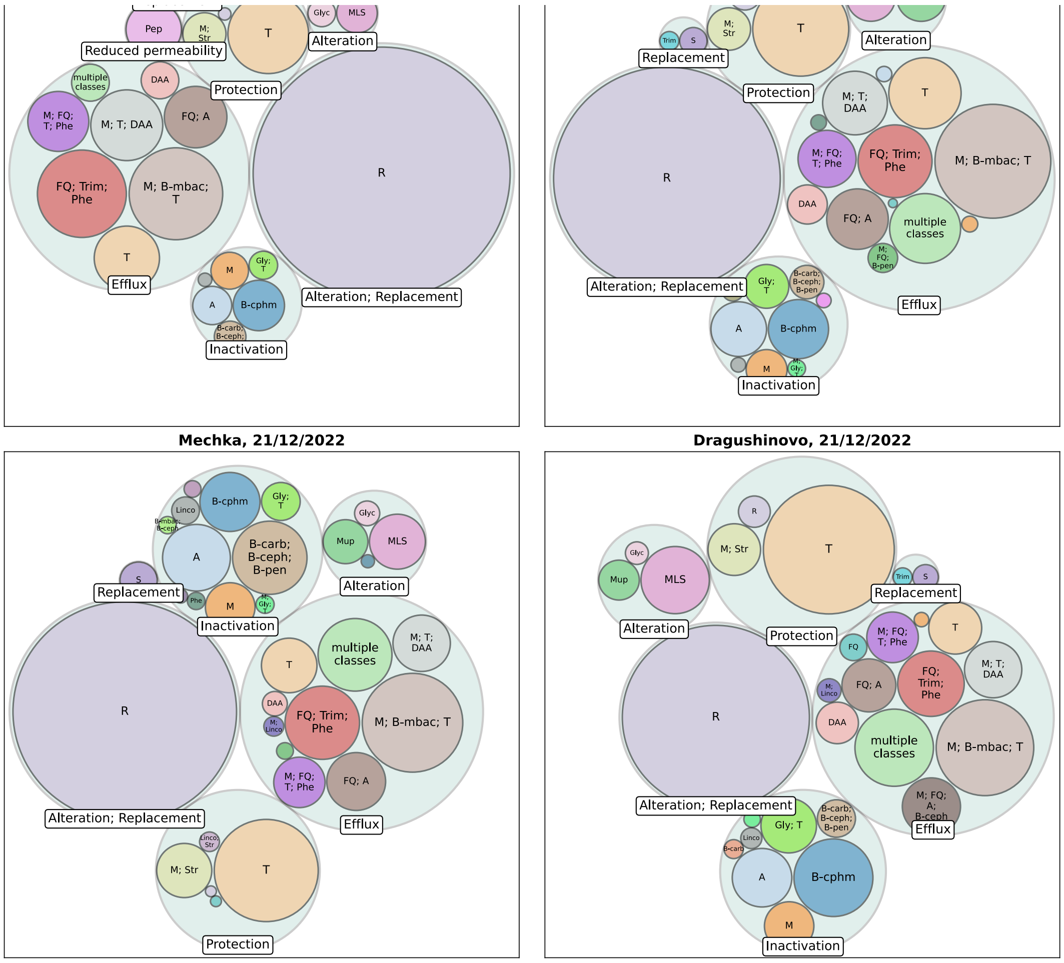

Gly - glycylcycline
Trim - diaminopyrimidine antibiotic
S - sulfonamide antibiotic
R - rifamycin antibiotic
Oxaz - oxazolidinone antibiotic
Phe - phenolic antibiotic
Fos - phosphonic acid antibiotic
Pep - peptide antibiotic
Mup - mupirocin-like antibiotic
Nitr - nitrofuran antibiotic
DAA - disinfecting agents and antiseptics
multiple classes - more than 4 classes

Supplementary Figure S6. Bubbleplot with antimicrobial resistance genes extracted from raw reads by location and date from CARD DB v3.2.7 with 60% coverage and 60% ID. Genes were grouped by drug class for general representation. Results from CARD DB were not considered and included in the Result section of the manuscript due to the high number of reported intrinsic genes.
